# Epitranscriptomic Modulation of TET2 Inhibition Suppressed SARS-CoV-2 Infection and Blocked Viral Nucleocapsid Protein in Induced-Pluripotent-Stem-Cell-Derived Cardiomyocyte Screening Models

**DOI:** 10.34133/bmr.0229

**Published:** 2025-07-22

**Authors:** Yi-Ping Yang, Chia-Hao Wang, Jun-Ren Sun, Yueh Chien, Chian-Shiu Chien, Guang-Yuh Chiou, Yun-Hsiang Cheng, Wen-Ting Chen, Ping-Cheng Liu, Shan-Ko Tsai, I-Hsun Chiang, Jui-Chia Wang, Huan Ou-Yang, Lo-Jei Ching, Wen-Liang Lo, Chien-Ying Wang, Hsin-Bang Leu, Chiu-Yang Lee, Shih-Hwa Chiou

**Affiliations:** ^1^ Department of Medical Research, Taipei Veterans General Hospital, Taipei, Taiwan.; ^2^ School of Medicine, National Yang Ming Chiao Tung University, Taipei, Taiwan.; ^3^ Institute of Pharmacology, School of Medicine, National Yang Ming Chiao Tung University, Taipei, Taiwan.; ^4^ Institute of Preventive Medicine, National Defense Medical Center, Taipei, Taiwan.; ^5^ Graduate Institute of Biodefense, National Defense Medical Center, Taipei, Taiwan.; ^6^ Graduate Institute of Applied Science and Technology, National Taiwan University of Science and Technology, Taipei, Taiwan.; ^7^ Division of Infectious Diseases and Tropical Medicine, Department of Internal Medicine, Tri-Service General Hospital, National Defense Medical Center, Taipei, Taiwan.; ^8^ Institute of Physiology, School of Medicine, National Yang Ming Chiao Tung University, Taipei, Taiwan.; ^9^ Department of Biological Science and Technology, National Yang Ming Chiao Tung University, Hsinchu, Taiwan.; ^10^ Department of Life Sciences and Institute of Genome Sciences, National Yang Ming Chiao Tung University, Taipei, Taiwan.; ^11^ Division of Oral and Maxillofacial Surgery, Department of Stomatology, Taipei Veterans General Hospital & National Yang Ming Chiao Tung University, Taipei, Taiwan.; ^12^ Department of Critical Care Medicine, Taipei Veterans General Hospital, Taipei, Taiwan.; ^13^ Division of Cardiology, Department of Medicine, Taipei Veterans General Hospital, Taipei, Taiwan.; ^14^ Healthcare and Management Centre, Taipei Veterans General Hospital, Taipei, Taiwan.; ^15^ Division of Cardiovascular Surgery, Department of Surgery, Taipei Veterans General Hospital, Taipei, Taiwan.; ^16^ National Yang Ming Chiao Tung University Hospital, Yilan, Taiwan.

## Abstract

Severe acute respiratory syndrome coronavirus 2 (SARS-CoV-2) viral infection has been associated with severe cardiovascular complications. However, the role of epitranscriptional modulation involved in SARS-CoV-2-infected myocarditis is still unclear. Ten-eleven translocation 2 (TET2), a methylcytosine dioxygenase, plays key roles in DNA demethylation during viral infection and host–virus interactions. Using human-induced-pluripotent-stem-cell-derived cardiomyocytes (hiPSC-CMs) as a platform, our data revealed the epitranscriptomic role of TET2 during SARS-CoV-2 infection. First, our RNA sequencing analysis revealed the alterations of the messenger-RNA-expression profiles of epitranscriptomic regulators, including TET2, in hiPSC-CMs during SARS-CoV-2 infection. Second, silencing TET2 markedly reduced both the messenger RNA and protein levels of the viral nucleocapsid (N) protein, leading to attenuated viral replication in infected hiPSC-CMs. Furthermore, RNA dot-blotting analysis revealed that TET2 knockdown suppressed the levels of 5-hydroxymethylcytosine in SARS-CoV-2-infected hiPSC-CMs. To further explore the therapeutic relevance of TET2 inhibition in suppressing SARS-CoV-2 infection, we screened and compared 3 structurally distinct TET2 enzymatic inhibitors: Bobcat339, TETi76, and TFMB-2HG. Among these, Bobcat339 demonstrated the most potent antiviral effect, markedly suppressing SARS-CoV-2 replication and N-protein expression. Molecular docking analysis revealed that Bobcat339 exhibited a high binding affinity for multiple viral targets, including nsp16, RdRp, and N protein, indicating a multitarget mechanism of action. In addition, our data demonstrated that treatment with Bobcat339 can suppress SARS-CoV-2 infectious activity and N-protein expression in infected hiPSC-CMs. Together, our findings highlight the regulatory role of TET2 in SARS-CoV-2 infection and identify Bobcat339 as a promising therapeutic compound. Understanding TET2-driven epitranscriptomics and the functions of TET-targeting inhibitors may provide a novel strategy for mitigating viral infection in SARS-CoV-2-induced cardiomyopathy.

## Introduction

The severe acute respiratory syndrome coronavirus 2 (SARS-CoV-2) pandemic has led to a surge in morbidity and mortality. Increasing amounts of clinical evidence reveal that SARS-CoV-2 not only affects the respiratory system but also damages multiple organs, resulting in conditions ranging from mild respiratory issues to life-threatening cardiovascular and pulmonary complications, including myocarditis, shock, and even sudden death [1]. Cardiomyocytes are known to overexpress angiotensin-converting enzyme 2 (ACE2), the receptor of SARS-CoV-2. In addition, cardiac damage and viral substances were observed in the autopsies of COVID-19 patients, indicating that SARS-CoV-2 may impair cardiomyocytes through mechanisms such as cytokine production, cell death, sarcomere disassembly, and contractile deficit [2]. The limited availability of physiologically relevant cardiac models has impeded the comprehensive understanding of these cardiac effects, especially in SARS-CoV-2-infected myocarditis and heart tissue damage [3]. While analyzing heart tissue samples could provide valuable insights into SARS-CoV-2-induced heart complications, the limited availability of such cardiac tissues poses a significant constraint on the scope of new drug development for emergent infectious diseases, including SARS-CoV-2.

Human induced pluripotent stem cells (hiPSCs) can be reprogrammed from somatic cells and possess the remarkable differential capability of multiple lineages [4]. One of the advantages of hiPSCs is that they do not require organ-tissue biopsy but can be differentiated into specific cell types to recapitulate various disease phenotypes [5–7]. By using hiPSC-derived models, researchers can explore disease mechanisms in vitro and gain detailed insights into the complex molecular and cellular signaling pathways involved, including SARS-CoV-2 infection [8]. For example, the use of hiPSC-derived lung organoids allows for the screening of potential antiviral drugs against SARS-CoV-2, leading to the identification of 2 flavonoid natural compounds [9]. Recent studies have used hiPSC-derived lung airway organoids as ex vivo alternative minitissues to study cytokine release and immune modulation during viral infections [6]. Notably, Zhang et al. [10] demonstrated that hiPSC-derived cardiomyocytes (hiPSC-CMs) exhibit functional similarities to their native human counterparts. It has been reported that iPSC-derived cardiomyocytes can endogenously express ACE2 and transmembrane protease serine 2 (TMPRSS2), providing an infectious model for studying the entry mechanism of SARS-CoV-2 [11]. Recent findings revealed that hiPSC-CMs are highly susceptible to the virus, resulting in altered gene expression and muscle fiber damage, mirroring observations in COVID-19 autopsy samples [5,8]. In addition, within the realm of viral infections, epigenetic and RNA modifications play crucial roles in mediating host–virus interactions and viral pathogenesis, especially during SARS-CoV-2 infection [12]. Even hiPSC-CMs have been successfully utilized as a research platform to explore the effects of SARS-CoV-2 infection; however, the entry mechanisms and epigenetic regulation of SARS-CoV-2-induced cardiomyopathy remain unclear. Clarifying the link between the epigenetic modifications of viral RNA genomes, including SARS-CoV-2, and viral replication and maturation could provide new insights and potential therapeutic strategies. Nevertheless, how to explore fundamental research and develop new drugs for targeting epigenetic and epitranscriptomic modulation in SARS-CoV-2-induced clinical complications and myocarditis is still unclear.

RNA modifications are prevalent posttranscriptional chemical alterations that are found across a diverse range of RNA molecules, including coding and noncoding RNAs [13]. These dynamic and reversible modifications regulate various biological processes, including RNA stability, messenger RNA (mRNA) translation, and gene expression. The dysregulation of 5-methylcytosine (m5C) modification and its associated regulators has been implicated in numerous diseases, including cancers, leukemia, and cardiovascular diseases [14]. With respect to viral infections, earlier findings reported that an m5C writer, NSUN2, regulates the epitranscriptomic modification during HIV infection [15]. Without the addition of m5C to the RNA transcripts by NSUN2, the production of the virus protein is reduced, which inhibits virus replication [16]. Ten-eleven translocation methylcytosine dioxygenase 2 (TET2), a member of the TET family of enzymes, is known to catalyze the hydroxylation of m5C marks within DNA, resulting in the generation of 5-hydroxymethylcytosine (hm5C) marks [17]. Importantly, Fu et al. [18] demonstrated that TET enzymes exhibited the activity of catalyzing the TET-mediated formation of hm5C in RNA in vitro. Shen et al. [19] demonstrated that TET2 exhibited posttranscriptional potential to promote pathogen-infection-induced myelopoiesis through mRNA oxidation. Furthermore, it has been reported that RNA-dependent chromatin targeting of TET2 and TET2-mediated RNA modification destabilized endogenous retrovirus in pluripotent stem cells [20]. Of note, the crucial evidence provided by Lan et al. [21] showed that the functional role of TET-mediated RNA hydroxymethylcytosine was involved in reducing the stability of crucial pluripotency promoting transcripts in mouse embryonic stem cells during differentiation. Nonetheless, the understanding of whether TET2-mediated epitranscriptomics and RNA modification mediate host–virus interactions and viral pathogenesis in SARS-CoV-2 infection could serve as potential therapeutic targets is limited.

It has been reported that epigenetic and epitranscriptomic modulation plays a key role in the pathogenic molecular mechanism involved in systemic viral infections and emerging infectious diseases [22]. Recently, small-molecule drugs that target RNA methylation have been extensively studied to improve the treatment efficacy for viral infections [23]. In this study, using hiPSC-CMs as an in vitro infectious myocarditis model for SARS-CoV-2 infection, we found that endogenous TET2 expression and TET2-mediated m5C hydroxylation were significantly up-regulated as part of the epitranscriptomic modifications following SARS-CoV-2 infection. Importantly, the gene knockdown of TET2 significantly reduced the level of hm5C RNA methylation, as detected by dot-blotting analysis. Consistently, TET2 inhibition resulted in significant antiviral activity, characterized by reduced viral nucleocapsid (N)-protein expression and markedly decreased SARS-CoV-2 infectivity in hiPSC-CMs. To identify potential therapeutic compounds targeting this pathway, we performed a comparative analysis of 3 representative TET2-related enzymatic inhibitors: Bobcat339, TETi76, and TFMB-2HG [24]. Using molecular docking simulations as the targeting platform for screening prediction, Bobcat339 exhibited a greater binding affinity for the TET2 protein than the other compounds did. Notably, western-blotting data revealed that Bobcat339 has the potential to significantly suppress SARS-CoV-2 viral replication and inhibit the expression of N protein. Furthermore, computational molecular docking simulations revealed strong binding affinities between Bobcat339 and 3 major SARS-CoV-2-encoded proteins: nonstructural protein 16 (nsp16), RNA-dependent RNA polymerase (RdRp), and N protein. In addition, TET2 knockdown suppresses SARS-CoV-2 infection by down-regulating N-protein expression and altering the level of hm5C and RNA modification. These findings highlight the importance of epitranscriptomic modulation in SARS-CoV-2-replicating infection, as well as targeting the TET2-mediated axis/pathways as a novel emergent viral-infection-based therapeutic strategy.

## Materials and Methods

### Generation and maintenance of hiPSC-CMs

**Fig. 1.**
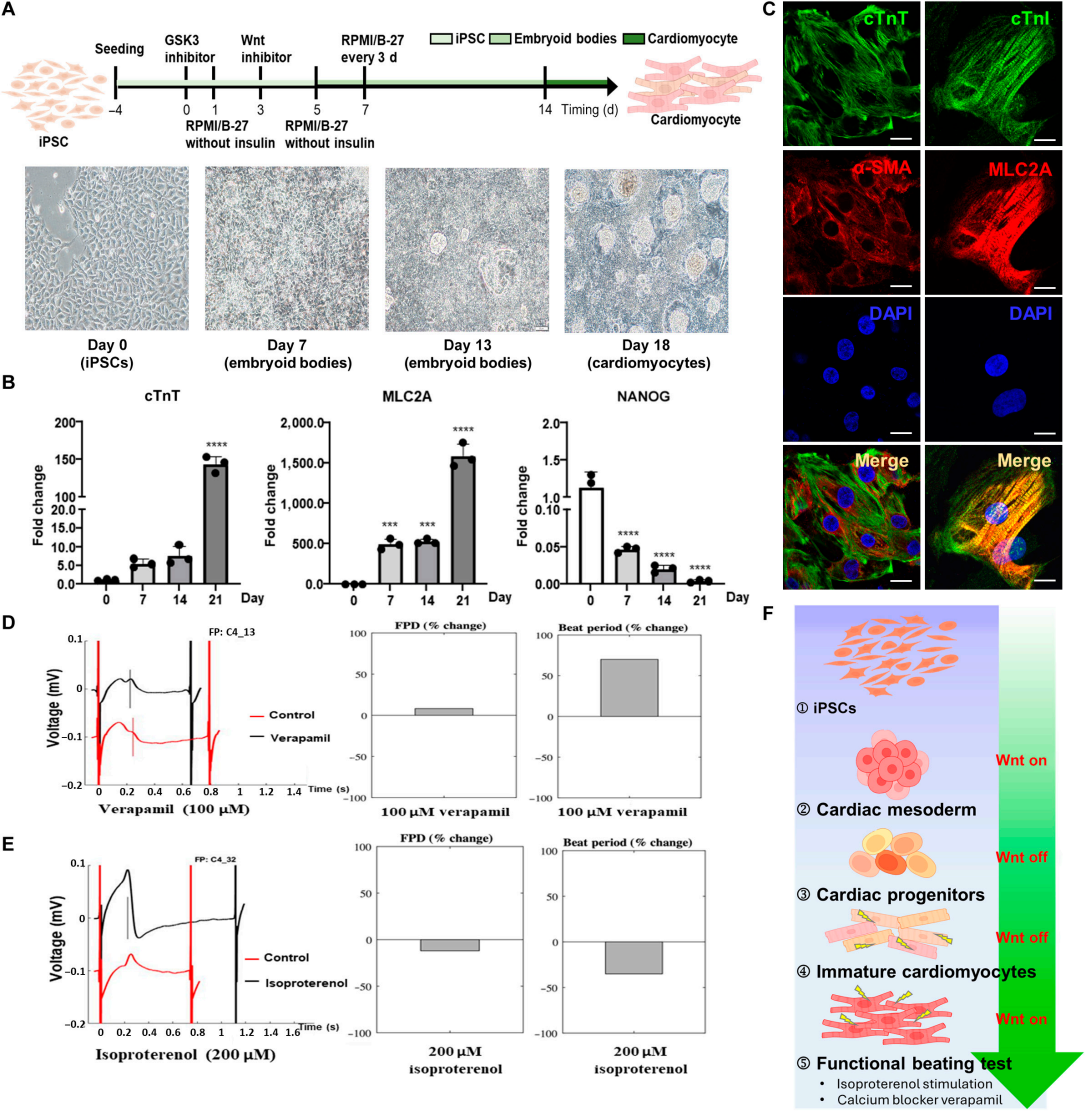
Establishment of human-induced-pluripotent-stem-cell-derived cardiomyocytes (hiPSC-CMs) as an in vitro experimental model and drug-responding platform. The hiPSC-CMs were successfully validated as an in vitro experimental model. (A) The schematic diagram illustrates the protocol of differentiation of hiPSCs into hiPSC-CMs. Bright-field images present the morphology of differentiated cells at the indicated time points. (B) The relative RNA expression levels of the indicated markers in the cells undergoing differentiation into hiPSCs at the indicated time points were identified via quantitative reverse transcription polymerase chain reaction (qRT-PCR). The bar charts represent the mean ± standard deviation (SD) of the relative results compared to those on day 0. Data are presented as mean ± SD, with significant differences indicated: ***P < 0.001 and ****P < 0.0001. (C) Fluorescence images from the immunofluorescent staining assay present cardiomyocyte markers in mature hiPSC-CMs. Scale bar: 20 μm. Microelectrode array recordings of field potential duration (FPD) and beat period of hiPSC-CMs in response to the application of pharmacological agents, (D) 100 μM verapamil and (E) 200 μM isoproterenol. (F) The schematic diagram illustrates the various cell stages and the activities of the Wnt signal in the differential process from iPSCs to mature cardiomyocytes. cTnT, cardiac troponin T; cTnI, cardiac troponin I; MLC2A, myosin regulatory light chain 2A; α-SMA, alpha-smooth muscle actin; DAPI, 4′,6-diamidino-2-phenylindole.

hiPSCs were cultivated for stable passages and stepwise differentiated into hiPSC-CMs following a protocol reported previously [11,16]. Geltrex-coated dishes (Thermo Fisher Scientific, Waltham, MA, USA) were preincubated at 37 °C for 24 h prior to hiPSC seeding with StemFlex medium (Thermo Fisher Scientific) supplemented with 10 μM rho-kinase inhibitor Y-27632 for 24 h after each passage. The cells were subcultured every 5 to 7 d when they reached 70% to 80% confluence [6]. For cardiomyocyte differentiation, hiPSCs were detached using Versene solution, plated onto 12-well plates, and cultured until they reached 60% to 70% confluence. On day 5, differentiation was initiated by adding a 1:3 ratio of old medium to RPMI/B27-insulin supplemented with 12 μM CHIR99021. After 24 h, the medium was replaced with RPMI/B27-insulin per well. On day 7, the medium was collected, mixed with fresh RPMI/B27-insulin containing 5 μM IWP2, and added back to the medium to inhibit Wnt signaling. From day 9, the medium was replaced every 3 d with RPMI/B27-insulin. The cultures were maintained in a 37 °C, 5% CO_2_ incubator, and robust spontaneous contractions were typically observed by day 14 [11]. The procedure is summarized in the workflow diagram shown in Fig. 1A.

### In vitro SARS-CoV-2 infection experiment

All experiments and manipulations with infectious SARS-CoV-2 were performed in appropriate biosafety level 3 (BSL-3) and BSL-4 laboratories, at the Institute of Preventive Medicine, National Defense Medical Center [6,11]. SARS-CoV-2 was obtained from the Taiwan Centers for Disease Control [6]. The study protocols used for the in vitro experiments involving SARS-CoV-2 infection followed previously published biosafety guidelines [6]. The SARS-CoV-2 strain used in this study was SARS-CoV-2/human/TWN/CGMH-CGU-15/2020 (MT374111.1), which was obtained from the National Defense Medical Center and propagated in Vero cells. Virus titers and culture supernatants from infected cardiomyocytes were quantified via plaque assays. The virus was diluted to the desired multiplicity of infection (MOI) in RPMI/B27 medium and incubated with hiPSC-CMs at 37 °C and 5% CO_2_ for 1 to 72 h. The control cells were treated with RPMI/B27 without infection. After exposure to the virus, the cells were washed 3 times with phosphate-buffered saline (PBS) and maintained in the appropriate culture medium.

### Tissue culture infectious dose (TCID_50_) assay

In accordance with previous protocols, for viral titer determination, hiPSC-CMs were infected for 1 h, after which the infection medium was replaced, and the supernatants were collected 24 h postinfection. These supernatants were subsequently used to infect confluent monolayers of Vero cells in 96-well plates. Cytopathic effects were evaluated visually 24 h after the infection of Vero cells. The infectious viral titer was calculated as the tissue culture infectious dose 50 (TCID_50_) per milliliter [6,11].

### Quantitative reverse transcription polymerase chain reaction

Following previous protocols [6], complementary DNA synthesis was carried out using SuperScript III Reverse Transcriptase (Thermo Fisher Scientific) with 500 to 1,000 ng of total RNA as input. Reverse transcription was performed using gene-specific forward and reverse polymerase chain reaction (PCR) primers for both negative-strand and positive-strand transcripts. Quantitative reverse transcription polymerase chain reaction (qRT-PCR) was conducted using SYBR Green and a real-time PCR system (Bio-Rad Laboratories, USA). The mRNA levels of each target gene in the samples were quantified via comparison with standard curves containing known concentrations ranging from 10^1^ to 10^6^ gene copies. The expression levels were normalized to those of GAPDH. Detailed information on the PCR primers used in this study can be found in Table S1.

### Immunofluorescence staining of hiPSC-CMs

Sterilized round #1.5 coverslips (13 mm in diameter, Thermo Fisher Scientific) were placed into the cell culture wells and coated with Geltrex overnight. hiPSC-CMs were then seeded onto the coated coverslips and incubated in the growth medium for 48 h. The cells were infected with viruses at MOIs ranging from 0.1 to 5 and incubated for 6 to 48 h. Following infection, the cells were fixed in 4% paraformaldehyde, washed with PBS, and permeabilized at room temperature with PBS containing 0.1% Triton X-100. The cells were then blocked in PBS containing 0.1% Tween 20 (PBST) containing 5% fetal bovine serum at room temperature, incubated overnight at 4 °C with primary antibodies diluted in the blocking solution, and subsequently incubated overnight at 4 °C with secondary antibodies diluted in blocking solution. 4′,6-Diamidino-2-phenylindole was then applied, and the samples were incubated overnight at 4 °C. Finally, the cells were washed with PBS, mounted onto slides with the mounting medium, and imaged using an Olympus confocal microscope (Olympus IX71, Olympus Corporation).

### Immunoblotting analysis

The cells were washed with ice-cold PBS and lysed using radioimmunoprecipitation assay lysis buffer (Visual Protein, Energenesis Biomedical, Taipei, Taiwan). To remove cellular debris, the lysates were centrifuged at 14,000 × g for 30 min at 4 °C, and the resulting supernatants were mixed with protein sample buffer and denatured at 95 °C for 5 min. The samples were loaded onto 6% to 12% sodium dodecyl sulfate–polyacrylamide gels and separated via electrophoresis. Following electrophoresis, the proteins were transferred onto polyvinylidene fluoride membranes (GE Healthcare, Chicago, IL, USA) and blocked with PBST containing nonfat milk for 1 h. The membrane was then incubated overnight at 4 °C with primary antibodies diluted in 5% blocking solution. After washing with PBST, the membrane was incubated with secondary antibodies diluted in 5% blocking solution for 2 h at room temperature. Following additional washes with PBST, Immobilon Western Chemiluminescent HRP Substrate was used to visualize the protein bands. Detailed information on the antibodies used in this study can be found in Table S2.

### Electrophysiological analysis

CytoView MEA plates (Axion BioSystems, Atlanta, GA, USA) were coated with Geltrex and seeded with hiPSC-CMs [11]. The effects of cardiotropic drugs on the electrophysiological properties of hiPSC-CMs were recorded using the Maestro Pro system and analyzed with the Cardiac Analysis Software v.3.1.8 (Axion BioSystems).

### RNA-seq analysis of SARS-CoV-2-infected hiPSC-CMs

The RNA sequencing (RNA-seq) libraries were prepared using GENEzol TriRNA Pure Kit (Geneaid, Taipei, Taiwan) according to the manufacturer’s protocol. Total RNA was extracted from infected cardiomyocytes, ensuring high purity and integrity suitable for sequencing. Library preparation included mRNA enrichment, fragmentation, complementary DNA synthesis, adapter ligation, and PCR amplification. The resulting libraries were quantified, validated for size distribution using Agilent Bioanalyzer, and pooled equimolarly for sequencing. Finally, the RNA-seq libraries were sequenced on an Illumina NovaSeq platform, generating high-throughput RNA-seq data for downstream determination and bioinformatics analysis [6,11].

### RNA dot-blotting assay

RNA was prepared by performing 2 consecutive 2-fold dilutions in RNase-free water. The RNA was then spotted onto a Hybond-N+ hybridization membrane and cross-linked to the membrane by ultraviolet irradiation. The membrane was blocked at room temperature for 1 h with 5% nonfat milk, followed by overnight incubation at 4 °C with a 1:1,000 dilution of anti-hm5C antibody (Active Motif, Carlsbad, CA, USA). After 3 washes, the membrane was incubated with the HRP-labeled peroxidase AffiniPure goat anti-rabbit IgG (Jackson ImmunoResearch Laboratories, West Grove, PA, USA). The membrane was then treated with Immobilon Western Chemiluminescent HRP Substrate (Merck Millipore, Burlington, MA, USA) and visualized via chemiluminescent detection [25].

### Liquid chromatography–mass spectrometry

RNA was isolated, and mRNA was extracted using Magnetic mRNA Isolation Kit (NEB #S1550). The mRNA samples were decapped with mRNA Decapping Enzyme for 1 h, followed by incubation overnight with nuclease P1 and dephosphorylation using alkaline phosphatase for 1 h. The samples were then diluted to a final volume of 100 μl with RNase-free water and filtered through a 0.2-μm filter. Analysis was conducted using the 6495C Triple Quadrupole LC/MS system (Agilent, CA, USA) with the Agilent MassHunter software [25]. The ratios of m5C and hm5C modifications relative to cytosine were calculated and normalized against a control sample [18].

### TET2 knockdown and cell viability assay

hiPSC-CMs were seeded at a density of 3 × 10^5^ cells per well in 12-well plates and transfected with either TET2-specific small interfering RNA (siRNA) or a scrambled sequence (control) for 48 h to achieve TET2 knockdown. The viability of TET2-knockdown hiPSC-CMs was assessed using alamarBlue reagent (Thermo Fisher Scientific). Following the transfection period, the culture medium was replaced with alamarBlue reagent, and the mixture was incubated for 1 h at 37 °C. The fluorescence intensity, which is indicative of cellular metabolic activity and viability, was measured using a Tecan Infinity 2000 plate reader (Tecan Group Ltd., Männedorf, Switzerland). The percentage viability was calculated relative to that of untreated cells, which were designated as 100% viability and included on each plate as a control reference.

### Treatment with the TET2 inhibitor Bobcat339

hiPSC-CMs were infected with SARS-CoV-2 at an MOI of 1 in RPMI medium supplemented with 2% B27. Prior to infection, the cells were treated with 20 to 80 μM of Bobcat339 (Selleck Chemicals, Houston, TX, USA) 24 h prior. The cells were harvested 24 h postinfection for subsequent RNA and protein analyses to investigate the effects of SARS-CoV-2 infection on cellular responses and molecular pathways in cardiomyocytes.

### Prediction of docking binding sites by molecular modeling simulation

The 3-dimensional structures of the target proteins were retrieved from the Protein Data Bank (PDB, https://www.rcsb.org/, accessed 9 Aug 2024), whereas the 3-dimensional structure of Bobcat339 was constructed using ChemSpider (https://www.chemspider.com/, accessed 10 Aug 2024). For docking simulations, the DockThor software (https://dockthor.lncc.br/v2/, accessed 10 Aug 2024) was used to prepare ligand and receptor files, adhering to previously published molecular docking protocols [26,27]. Redocking was conducted for TET2 and virus-associated proteins, with grid parameters defined to center coordinates (*X*, *Y*, *Z*) and a grid size of 20 Å, as established by the LibDock method. The docking simulations were performed using the DockThor standard search algorithm, with 12 independent runs executed for each target–ligand pair. The docking results were further refined using the Discovery Studio 2.1 software, which employs the smart minimize algorithm and CHARMm force field for energy minimization. To further evaluate the binding affinity of Bobcat339 for virus-associated proteins, GEMDOCK (The Institute of Bioinformatics, National Chiao Tung University, Hsinchu, Taiwan) was utilized. The binding site of each virus-associated protein was defined based on known interaction regions, and docking simulations were carried out to calculate the binding energy, providing insights into the specificity and potential multitarget activity of Bobcat339.

### Statistical analyses

The data are presented as mean ± standard error of the mean (SEM). Details regarding the type and number of replicates, the specific statistical analyses performed, the statistical tests employed, and the corresponding results are provided in the figure legends accompanying each dataset. All statistical analyses were conducted using the Prism software, with the significance level set at *P* < 0.05.

## Results

### Establishment of hiPSC-derived cardiomyocytes as a drug-responding screening platform

Given the challenges associated with acquiring functional human cardiomyocytes for experimental use, a range of model systems have been employed to study cardiac diseases, including primary human cardiomyocytes [9,10]. However, the high risk associated with the use of clinical heart biopsy for cultivating primary cardiomyocytes is still limited [28]. Therefore, an alternative and safe approach is to utilize a hiPSC-differentiated cardiomyocyte (hiPSC-CM) model to overcome these challenges. Previously, we established a protocol for generating functional hiPSC-CMs as an in vitro cardiac disease model and drug screening platform [11]. In this study, we utilized a hiPSC-CM platform, which represents a powerful model for drug screening and identification of therapeutic targets against SARS-CoV-2 infection in the heart. The protocols used to cultivate hiPSC-based heart-lineage and differentiated cardiomyocytes are as follows: Briefly, the temporal modulation of canonical Wnt signaling promotes cardiac mesoderm formation [11], inhibits it during the cardiac progenitor stage, and reactivates it to mature cardiomyocytes (Fig. 1A). The qualities and maturities of our cultivated hiPSC-CMs were characterized via qRT-PCR and immunostaining to evaluate the mRNA and protein expression levels of cardiac marker proteins. Compared with those of hiPSCs (undifferentiated cells), the results of qRT-PCR revealed that these hiPSC-differentiated cells highly expressed cardiomyocyte markers, such as cardiac troponin T (cTnT; Fig. 1B) and myosin regulatory light chain 2A (MLC2A; Fig. 1B), whereas the expression of NANOG, a pluripotent stem cell marker, was markedly decreased (Fig. 1B). Furthermore, the endogenous protein signals of cTnT and MLC2A, both of which are markers of sarcomeres and thin filaments of heart muscle, were highly expressed and colocalized in the same differentiated hiPSC-CMs via immunostaining analysis (Fig. 1C). The results of qRT-PCR and immunofluorescent imaging confirmed that the hiPSCs were successfully differentiated into mature cardiomyocytes.

Moreover, the functionality of hiPSC-CMs was further assessed via the analysis of electrophysiological experiments. Electrophysiological responses with sharp microelectrode recordings were detected on spontaneously contracting hiPSC-CMs. Treatment of iPSC-CMs with verapamil resulted in an increased field potential duration (Fig. 1D), while isoproterenol decreased the field potential duration (Fig. 1E). These results indicated that hiPSC-CMs present mature and well-functioning cardiomyocytes that efficiently respond to the individual therapeutic effects of verapamil and isoproterenol treatment, indicating the positive and negative chronotropic effects (Fig. 1D and E). Collectively, we demonstrated that hiPSC-CMs presented a functional cardiac microenvironment with therapeutic effects, serving as an in vitro drug-responsive and screening model for investigating the cellular mechanisms underlying their pharmacologic effects on the heart (Fig. 1F).

### Using hiPSC-CMs as an in vitro SARS-CoV-2-infected target for new drug screening and development

**Fig. 2.**
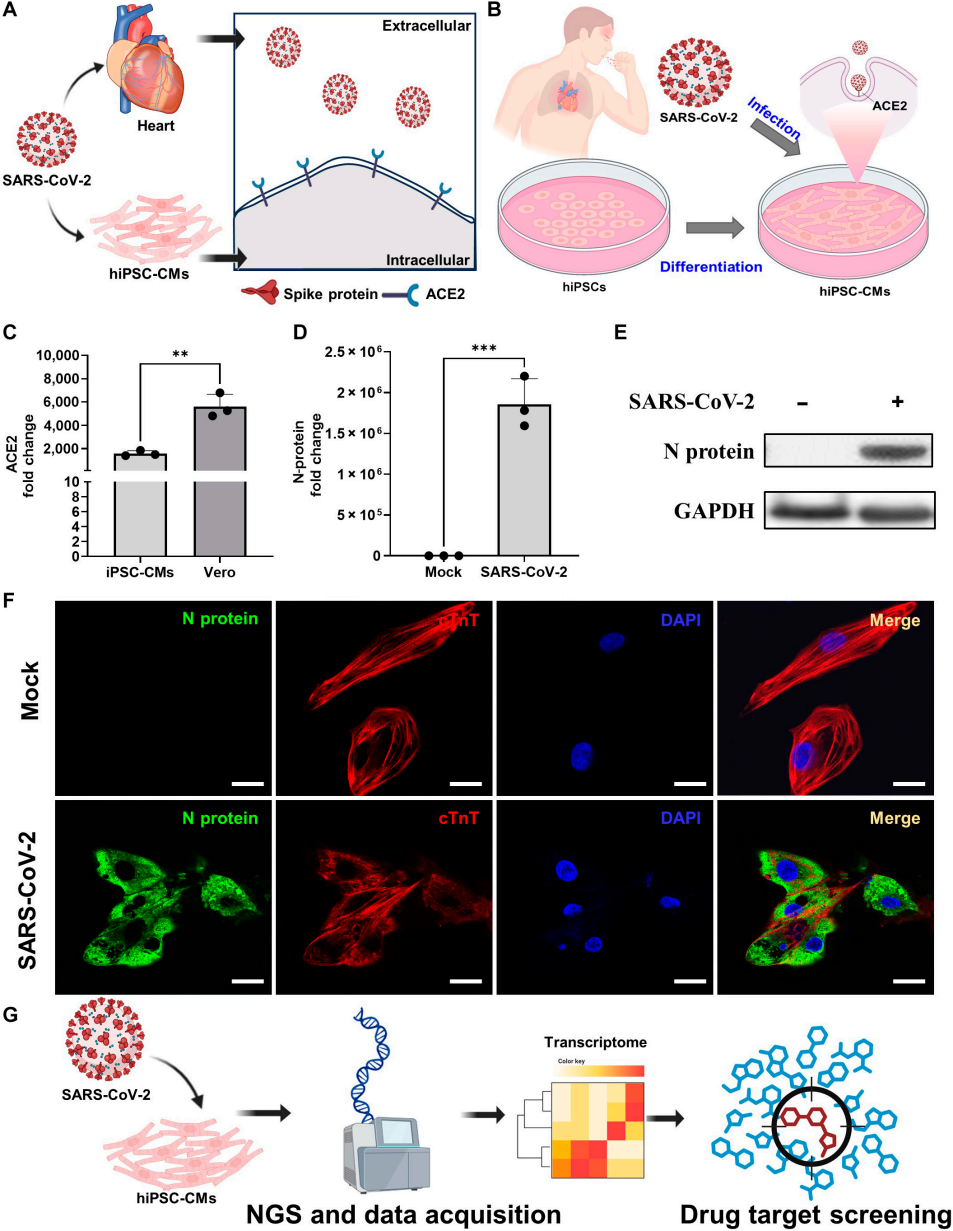
Using hiPSC-CMs as an in vitro severe acute respiratory syndrome coronavirus 2 (SARS-CoV-2)-infected target for new drug screening. (A) The schematic diagram illustrates that SARS-CoV-2 can directly target the heart and hiPSC-CMs via the interactions between viral spike protein and the host’s angiotensin-converting enzyme 2 (ACE2) protein (B) The schematic diagram illustrates the protocol of SARS-CoV-2 infection assay in the in vitro drug screening hiPSC-CM model. (C) Relative gene expression levels of ACE2 were calculated using the ΔΔCT method via qRT-PCR, expressed as fold change relative cells. (D) Bar graph representing the relative gene expression levels of viral nucleocapsid (N) protein calculated using the ΔΔCT method via qRT-PCR. Data are expressed as fold change relative to the mock group. Data are presented as mean ± SD, with significant differences indicated: **P < 0.01, ***P < 0.001, and ****P < 0.0001. (E) Western blot analysis shows the protein enrichment of N proteins in hiPSC-CMs infected with SARS-CoV-2. GAPDH is used as the loading control. (F) Fluorescence images show the spatial distribution of N protein (green) and cTnT (red) within hiPSC-CMs under conditions of the absence or presence of SARS-CoV-2 infection. Nuclei are stained with DAPI (blue). The merged images represent the presence of the N protein in the infected mature hiPSC-CMs, cTnT-positive cells. Scale bar: 20 μm. (G) The illustration details the flow of drug target screening. NGS, next-generation sequencing.

The roles of epigenetics in the regulation of SARS-CoV-2 entry and immunomodulation during infection represent a potential target for the development of anti-SARS-CoV-2 drugs [29]. However, the specific epigenetic mechanisms contributing to various aspects of SARS-CoV-2 pathogenesis remain unclear. ACE2 and TMPRSS2 are 2 major entry receptors for SARS-CoV-2 infection (Fig. 2A), and the cellular entry of SARS-CoV-2 in infected host cells represents potential targets for drug development [30]. Our previous studies revealed that hiPSC-CMs endogenously expressed ACE2 and TMPRSS2, which are 2 critical proteins that facilitate SARS-CoV-2 infection entry [11]. Based on the findings that hiPSC-CMs present a functional cardiac environment with drug-responsive features (Fig. 1), we further investigated whether these hiPSC-CMs are susceptible to infection by SARS-CoV-2 and can facilitate entry into host cells through the endocytosis pathway (Fig. 2B). Consistent with our previous observations, qRT-PCR analysis confirmed that both ACE2 and TMPRSS2 were expressed in hiPSC-CMs, indicating the presence of essential host factors for SARS-CoV-2 entry (Fig. 2C). Vero cells, which served as a positive control, presented high ACE2 expression, and TMPRSS2 expression was validated separately. We next investigated the susceptibility of hiPSC-CMs to SARS-CoV-2 infection. At 24 h postinfection with an MOI of 1, qRT-PCR analysis revealed that the viral N gene was robustly up-regulated in hiPSC-CMs (Fig. 2D), indicating efficient viral entry and replication. Furthermore, we evaluated the differences in the viral infectivity and replication between hiPSC-CMs and human cardiomyocyte cells coexpressing ACE2 and TMPRSS2 (Fig. S1; AC-16-coexpressed ACE2/TMPRSS2 [30]). Importantly, western blot analysis confirmed a pronounced increase in N-protein expression in infected hiPSC-CMs at the same time point (Fig. 2E), providing strong protein-level evidence of productive viral replication in this in vitro cardiac model. Moreover, the N protein of SARS-CoV-2 can be significantly observed in hiPSC-CMs 24 h postinfection by immunofluorescence staining, and signals of cTnT proteins (red fluorescence: mature cardiomyocyte marker) were highly costained with the N protein of the infected hiPSC-CMs (Fig. 2F). Based on these in vitro viral infection experiments, our data suggest that hiPSC-CMs serve as a potential infection-targeting model of SARS-CoV-2 infection. In summary, we demonstrated that hiPSC-CMs are a suitable in vitro model for mimicking SARS-CoV-2 infection of heart tissues, and this model has potential as an in vitro SARS-CoV-2 infection model for drug screening and development.

### Alterations in the mRNA expression of epitranscriptomic regulators in hiPSC-CMs during SARS-CoV-2 infection

**Fig. 3.**
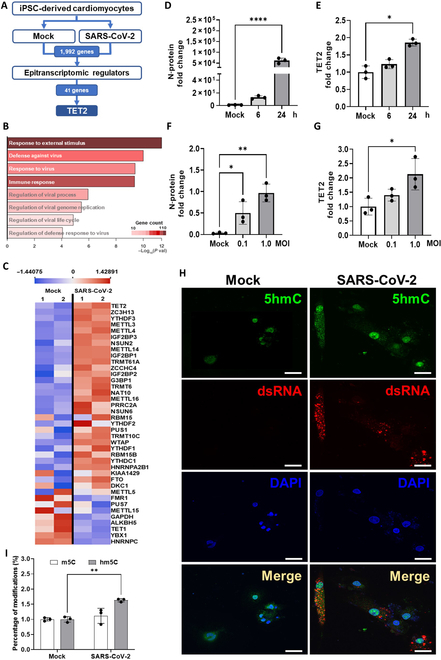
Alterations in the messenger RNA (mRNA) expression of epitranscriptomic regulators by RNA sequencing (RNA-seq) in hiPSC-CMs during SARS-CoV-2 infection. (A) Schematic diagram illustrating the workflow for comparing gene expression profiles between uninfected (the “mock” group) and SARS-CoV-2-infected iPSC-derived cardiomyocytes. (B) Gene Ontology biological process (GO-BP) enrichment analysis of up-regulated pathways in SARS-CoV-2-infected hiPSC-CMs, based on RNA-seq data. (C) Heatmap showing the expression levels of ten-eleven translocation 2 (TET2) and other RNA modification regulators in control hiPSC-CMs (mock) and infected hiPSC-CMs. Two biological replicates per group are shown, ranked from top to bottom by fold change in gene expression. (D and E) Expression levels of SARS-CoV-2 N protein and TET2 in hiPSC-CMs infected with different multiplicities of infection (MOIs; 6 to 24 h). (F and G) Quantification of the expression levels of SARS-CoV-2 N protein and TET2 in hiPSC-CMs infected with different MOIs (0.1 to 1). (H) Fluorescence images showing the spatial distribution of 5-hydroxymethylcytosine (hm5C; green) and double-stranded RNA (dsRNA; red) in hiPSC-CMs with or without SARS-CoV-2 infection. Nuclei are stained with DAPI (blue). Scale bar: 20 μm. (I) Quantification of modified nucleosides in infected and noninfected hiPSC-CMs by mass spectrometry. The statistical significance between groups (mock vs. SARS-CoV-2-infected groups) is denoted by asterisks: *P < 0.05, **P < 0.01, ***P < 0.001, and ****P < 0.0001. m5C, 5-methylcytosine.

Understanding the molecular pathways of SARS-CoV-2-derived viral effects is crucial for developing targeted treatments. High-throughput genome-wide sequencing and large-scale screening profiling of mRNA expression have been shown to be key strategies for identifying novel biomarkers and therapeutic targets [31]. To decipher the transcriptional alterations and the complex interactions between SARS-CoV-2 and infected hiPSC-CMs, we performed RNA-seq analysis on the cells subjected to infection at an MOI of 1, which were sampled 24 h postinfection. A total of 1,992 genes presented different expression patterns between the mock and SARS-CoV-2 infection groups. Among the 41 epitranscriptomic/RNA modification regulator-encoding genes, several genes presented differences in expression levels following SARS-CoV-2 infection (Fig. 3A). The results revealed that the most up-regulated pathways included those involved in viral defense, the regulation of viral processes, viral genome replication, and the immune response (Fig. 3B). Notably, the results of RNA next-generation sequencing with bioinformatics analysis demonstrated that the transcriptomic expression profiles of epigenetics-related genes—including TET2, METTL3, METTL4, METTL14, METTL16, YTHDF1, WTHDF2, WTAP, and FTO—were markedly altered post-SARS-CoV-2 infection when compared with those of the control (Fig. 3C). These genes exhibited significant changes in expression patterns, suggesting their potential involvement in the cellular response to SARS-CoV-2 in infected cardiomyocytes.

To further investigate whether TET2 expression levels were correlated with infection duration and viral load, we analyzed the mRNA-expression levels of N protein (a marker of SARS-CoV-2; Fig. 3D) and TET2 (Fig. 3E) in hiPSC-CMs 6 and 24 h postinfection. The mRNA-expression levels of both TET2 and N protein significantly increased after infection. Furthermore, when hiPSC-CMs were infected with increasing MOIs of 0.1 and 1, we observed an increase in the mRNA-expression levels of both the N protein (Fig. 3F) and TET2 mRNAs (Fig. 3G). Moreover, double-stranded RNA (dsRNA), a key indicator of active viral replication in SARS-CoV-2 infection, has been demonstrated to visualize the active viral RNA synthesis and replication in infected cells via immunofluorescence staining [32]. To further explore the protein-level profile of SARS-CoV-2 infection and epitranscriptomic markers, the technological platform of confocal fluorescence microscopy was used to detect the expression levels and localization of hm5C, as well as the expression of infectious markers in mock (noninfectious) and infected iPSC-CMs (Fig. 3H). Our results revealed that the protein expression of hm5C contained dsRNA antibodies (a protein marker of viral replication of SARS-CoV-2) in the same infected hiPSC-CMs (Fig. 3H). Notably, compared with those in mock control group, the expression levels of hm5C and dsRNA were significantly increased with a diffuse pattern and discrete cytosolic puncta in infected cells (Fig. 3H). These results suggest that SARS-CoV-2 infection may alter hm5C expression and abundance, potentially reflecting changes in RNA epigenetic regulation during infection. In addition, our liquid chromatography–mass spectrometry analysis confirmed that the levels of hm5C were significantly increased 24 h post-SARS-CoV-2 infection compared with those in the control group (mock infection; Fig. 3I). Taken together, our findings indicate that SARS-CoV-2 infection is involved in the epitranscriptomic modulation of TET2 expression in hiPSC-CMs, suggesting that TET2-driven epitranscriptomic targets play some roles in the context of SARS-CoV-2-induced cardiomyopathy.

### TET2 knockdown suppressed viral replication and N-protein expression during SARS-CoV-2 infection

**Fig. 4.**
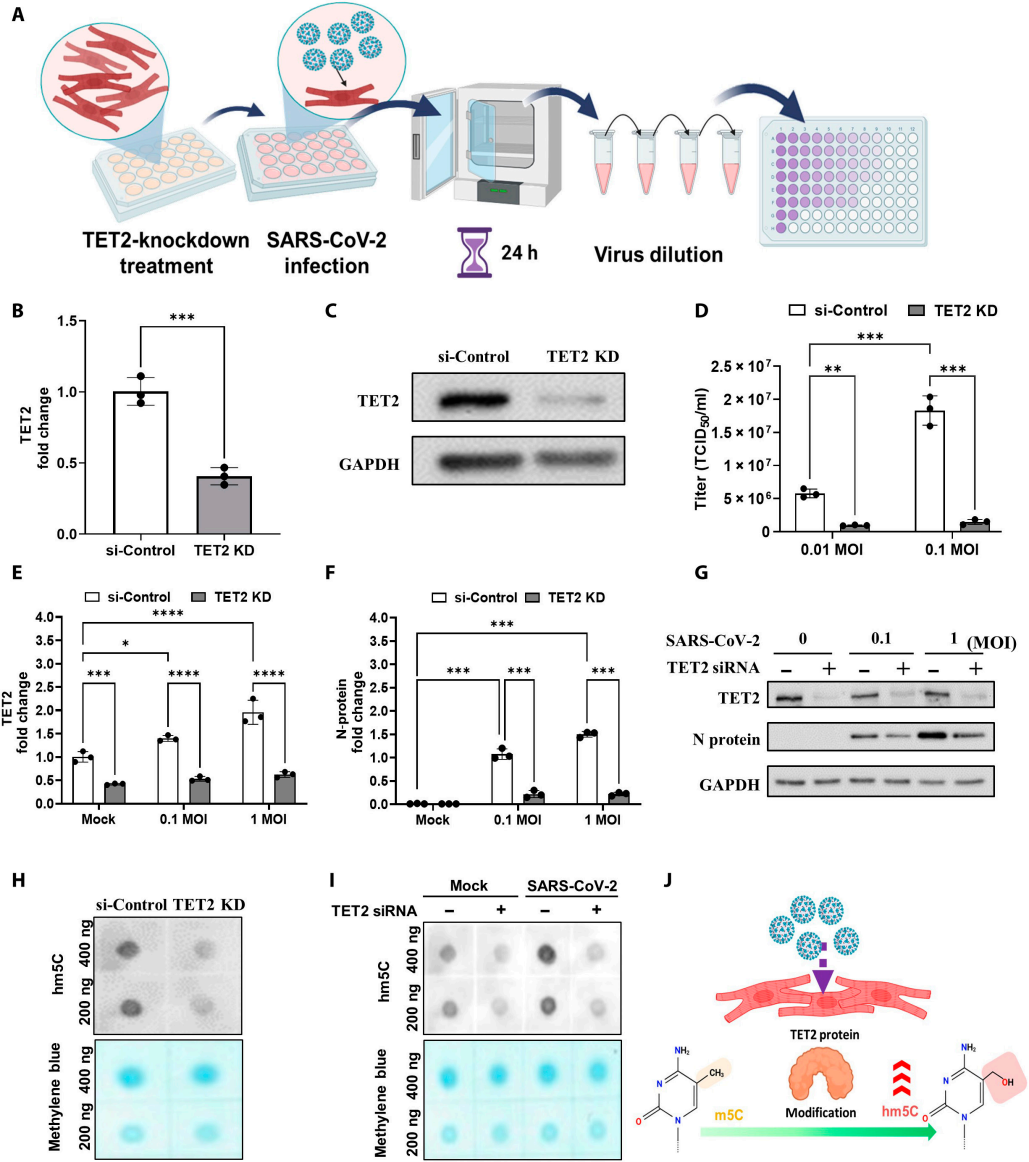
TET2 knockdown suppressed SARS-CoV-2 replication and N-protein expression in infected hiPSC-CMs. (A) Schematic diagram illustrating the viral infection (tissue culture infectious dose 50 [TCID_50_]) assay workflow in hiPSC-CMs with TET2 knockdown treated with si-TET2 small interfering RNA (siRNA). (B) Relative gene expression of TET2 wase performed via qRT-PCR at 48 h post-transfection with either si-Control or si-TET2. Data are presented as mean fold change relative to the scrambled control (si-Control) with SD error bars, showing a significant reduction in TET2 expression after knockdown (***P < 0.001). (C) Western blot analysis showing the protein levels of TET2 in hiPSC-CMs following TET2 knockdown. (D) Quantification of infectious SARS-CoV-2 titers in the medium from hiPSC-CMs subjected to TET2 knockdown and infected with SARS-CoV-2 at the indicated MOI. Data are presented as mean ± SD, with significant differences indicated as follows: **P < 0.01 and ***P < 0.001. (E and F) qRT-PCR quantification of fold changes in the mRNA levels of TET2 (E) and N protein (F) under various MOIs, calculated using the ΔΔCT method. (G) Western blot analysis comparing TET2 and N-protein levels across different SARS-CoV-2 MOIs, with or without si-TET2 treatment. (H) RNA dot blot analysis of hm5C levels in infected hiPSC-CMs with or without si-TET2 treatment. Total RNA was stained with methylene blue. (I) Dot blot analysis of hm5C levels in the total RNA of hiPSC-CMs infected with SARS-CoV-2 with or without si-TET2 treatment. Total RNA is stained with methylene blue. (J) Diagram illustrating the TET2-catalyzed oxidation of m5C to hm5C. KD, knockdown.

TET2 is an enzyme involved in the epigenetic regulation of gene expression through its role in DNA demethylation [17]. Recent studies have revealed that TET2 can also catalyze the hydroxylation of m5C marks within RNA, resulting in the epitranscriptomic generation of hm5C marks in controlling normal cellular functions and stem cell differentiation [21]. However, the roles of TET2 involved in the biomolecular mechanism to epitranscriptomically modulate the viral replication of SARS-CoV-2 infection are still unclear. As shown in Fig. 3, our data indicate that SARS-CoV-2 infection was associated with the mRNA expression of TET2 and epitranscriptomic regulators. To explore the potential role of TET2 in the biomolecular mechanism and epitranscriptomic modulation of SARS-CoV-2 infection, TET2 expression was silenced using si-TET2 siRNA in SARS-CoV-2-infected hiPSC-CMs and the control group (Fig. 4A). As shown in Fig. 4B, treatment with si-TET2 siRNA significantly reduced TET2 mRNA expression, as determined via qRT-PCR (Fig. 4B). Compared with the control, siRNA-mediated knockdown of TET2 resulted in a significant decrease in TET2 protein levels in treated hiPSC-CMs (Fig. 4C). To further investigate whether TET2 expression interferes with SARS-CoV-2 infection, TET2 knockdown largely suppressed viral infection (TCID_50_) in treated hiPSC-CMs 24 h postinfection (Fig. 4D). Next, considering the crucial function of the SARS-CoV-2 N protein in viral genome packaging and virion assembly, we further examined the efficacy of TET2-siRNA-mediated knockdown of viral replication and N-protein expression. Compared with those in the control groups 24 h postinfection, the qRT-PCR results revealed that the endogenous mRNA-expression levels of TET2 (Fig. 4E) and N protein (Fig. 4F) were significantly lower in the si-TET2-treated groups at 0.1 and 1 MOIs of SARS-CoV-2 infection, respectively (Fig. 4E and F). These data indicated that TET2 knockdown not only markedly blocked the expression of the N protein but also significantly suppressed the ability of SARS-CoV-2 to infect hiPSC-CMs (Fig. 4D to F). Notably, western blot analysis revealed that treatment with siRNAs to knock down TET2 also significantly decreased N expression at the protein level, even under high viral titers (0.1 and 1 MOI of SARS-CoV-2-infected conditions, respectively) (Fig. 4G).

We further investigated the potential roles of TET2 involved in the epitranscriptomic modulation in infected hiPSC-CMs during SARS-CoV-2 infection. First, the results of RNA dot-blot analysis revealed that the level of hm5C was markedly reduced following TET2 knockdown in iPSC-CMs (Fig. 4H). Furthermore, we explored the roles of TET2 in modulating hm5C and RNA modification during SARS-CoV-2 infection. Compared with those in the mock control, the expression levels of hm5C were significantly increased in infected hiPSC-CMs (Fig. 4I). Notably, the levels of hm5C were markedly suppressed by TET2-knockdown treatment in SARS-CoV-2-infected hiPSC-CMs (Fig. 4I). Collectively, these results demonstrated that SARS-CoV-2 infection led to increased RNA hydroxymethylation, and knockdown of TET2 may effectively suppress viral infection and N-protein expression, as well as inhibit the level of hm5C during SARS-CoV-2 infection (Fig. 4I). In addition, the roles of TET2 in the epitranscriptomic regulation of RNA modification during SARS-CoV-2 infection need to be further studied in the future (Fig. 4J).

### Screening candidates for TET-family inhibitors for TET2-protein-binding activity via computational molecular docking simulation

**Fig. 5.**
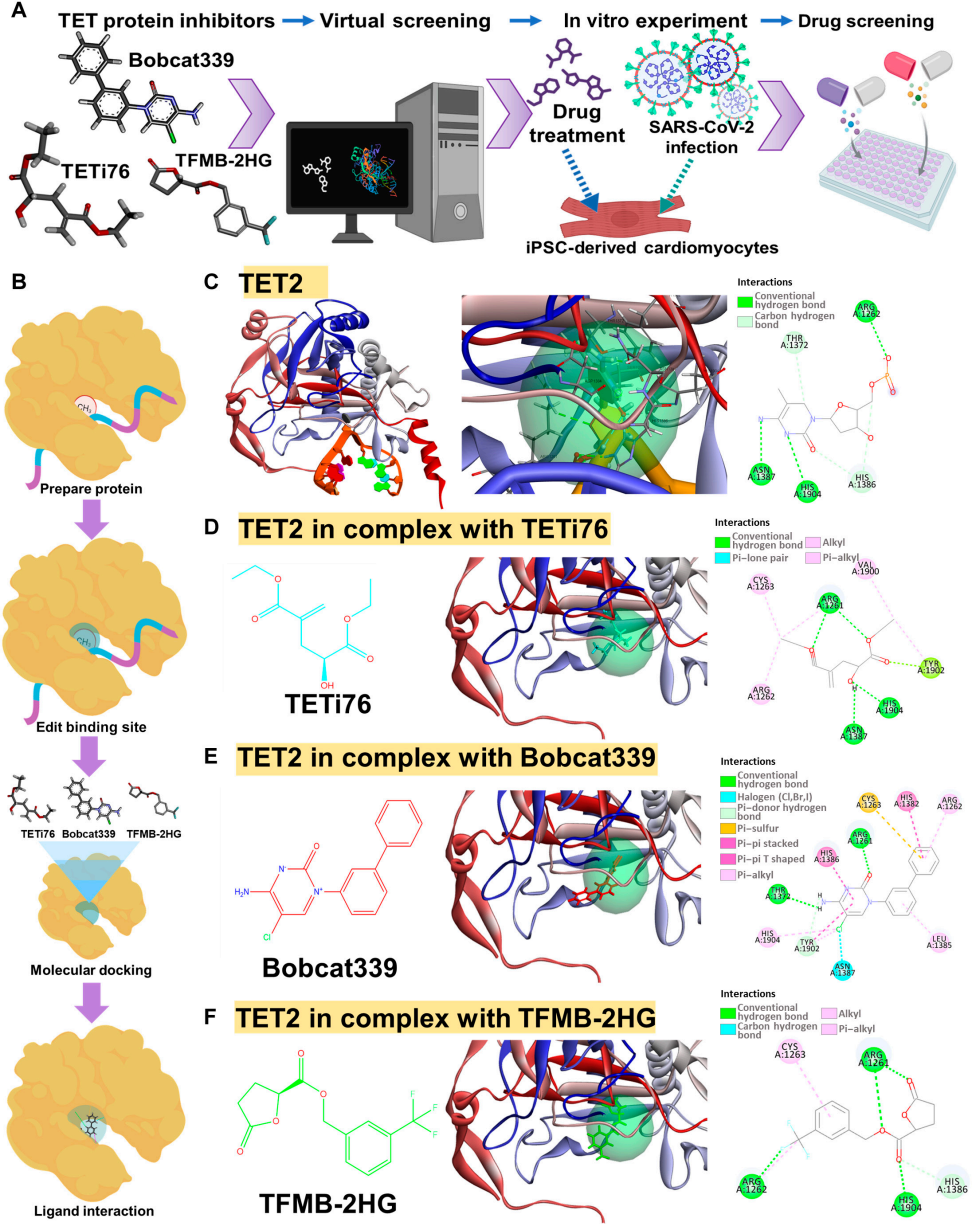
Screening potential candidates of TET-family inhibitors in infected hiPSC-CMs via computational molecular docking simulation. (A) The flowchart of the drug screening platform for molecular docking analysis of TET2 in complex with its DNA substrate and representative inhibitors. (B) Schematic representation of the molecular docking of representative TET2 inhibitors—Bobcat339, TFMB-2HG, and TETi76—into the catalytic pocket of TET2. The figure illustrates the catalytic core of TET2 binding to methylated DNA, followed by computational screening and docking of small-molecule inhibitors into the active site. (C) TET2 in complex with DNA substrate (Protein Data Bank [PDB] ID: 7NE3). Left: Overall structure showing TET2 (ribbon representation) bound to DNA (sticks in orange and magenta). Middle: Close-up view of the catalytic pocket highlighting the interaction surface. Right: 2-dimensional (2D) interaction diagram illustrating contacts between TET2 and the DNA substrate. (D) TET2 in complex with TETi76. Left: Chemical structure of TETi76. Middle: Docked pose of TETi76 in the TET2 catalytic site. Right: 2D interaction diagram showing hydrogen bonding and hydrophobic contacts. (E) TET2 in complex with Bobcat339. Left: Chemical structure of Bobcat339. Middle: Docked binding mode of Bobcat339 in the catalytic site. Right: 2D interaction map illustrating hydrogen bonds, hydrophobic interactions, and halogen bonding. (F) TET2 in complex with TFMB-2HG. Left: Chemical structure of TFMB-2HG. Middle: Docked pose within the TET2 active site. Right: 2D interaction map showing hydrogen bonding and minimal hydrophobic interactions.

Since the inhibition of TET2 and TET2-related functions suppresses infectious activity and viral replication during SARS-CoV-2 infection, we screened potential TET-family inhibitors as candidates for anti-SARS-CoV-2 therapeutics. To evaluate the functional targeting efficacy and protein-binding affinity of these TET-family inhibitors, we employed computational molecular docking prediction as a drug screening platform focused on TET2 inhibition. The overall workflow of our screening strategy is illustrated in Fig. 5A, where compounds with the highest predicted binding affinity to the TET2 catalytic pocket were first identified through in silico modeling, followed by functional validation using a cellular assay platform to evaluate their inhibitory efficacy. Three candidate TET-family inhibitors—TETi76, TFMB-2HG, and Bobcat339 [24]—were evaluated via molecular docking simulation. As shown in Fig. 5B, the chemical structures of the 3 selected inhibitors are displayed alongside a schematic diagram illustrating the overall docking strategy, in which each compound was computationally positioned within the TET2 catalytic pocket for interaction profiling. Docking simulations were performed using the crystal structure of TET2 in complex with its DNA substrate (PDB ID: 7NE3), with the catalytic pocket defined by key interacting residues such as ARG1262, THR1372, HIS1386, ASN1387, and HIS1904 (Fig. 5C). The 3 compounds demonstrated distinct interaction profiles with the TET2 active site. TETi76 formed hydrogen bonds with ARG1261, LEU1387, and HIS1904 and established hydrophobic contacts with CYS1262, ARG1263, VAL1900, and TYR1902 (Fig. 5D).

Bobcat339 interacted with multiple residues via hydrogen bonding (e.g., ARG1261, THR1372, and TYR1902), hydrophobic contacts (e.g., ARG1262, CYS1263, HIS1382, LEU1385, HIS1386, and HIS1904), and a halogen bond with ASN1387 (Fig. 5E). TFMB-2HG primarily engaged in hydrogen bonding with ARG1261, ARG1262, HIS1386, and HIS1904, along with limited hydrophobic interactions (Fig. 5F). Quantitative analysis via DockThor and iGEMDOCK revealed that Bobcat339 exhibited a relatively higher predicted binding affinity (DockThor: −7.968 kcal/mol; iGEMDOCK total interaction energy: −101.03 kJ/mol), with major contributions from van der Waals interactions (−82.41 kJ/mol) and hydrogen bonding (−18.62 kJ/mol) (Table 1). To validate our computational predictions, we assessed the anti-SARS-CoV-2 potential of these TET-family inhibitors by evaluating their ability to interfere with viral infection using the TCID_50_ assay. Compared with the untreated control group, all 3 compounds—TETi76, TFMB-2HG, and Bobcat339—demonstrated inhibitory effects on viral infection in hiPSC-CMs at 24 h post-SARS-CoV-2 infection (Fig. S2). Based on these findings, Bobcat339 was selected for subsequent experimental investigations to further explore its therapeutic potential in the context of SARS-CoV-2 infection.

**Table 1.** Chemical energy calculated by DockThor and iGEMDOCK for the interaction between TET2 inhibitors and the binding pocket of the TET2 protein. For docking simulations, (a) the DockThor software (https://dockthor.lncc.br/v2/, accessed 10 Feb 2024) was used to prepare ligand and receptor files, adhering to previously published molecular docking protocols, and (b) iGEMDOCK provided complementary docking analysis, offering detailed interaction energies including hydrogen bond contributions.

Compound ^a^	(a) DockThor				(b) iGEMDOCK		
	Affinity (kcal mol^−1^)	Total energy (kcal mol^−1^)	VDW energy (kcal mol^−1^)	Elec. energy (kcal mol^−1^)	Total energy (kJ mol^−1^)	VDW energy (kJ mol^−1^)	H bond (kJ mol^−1^)
Bobcat339	−7.968	38.928	−8.901	−7.360	−101.03	−82.41	−18.62
TFMB-(S)-2-HG	−7.331	11.252	−19.685	−7.658	−95.12	−74.07	−21.05
TETi76	−6.523	3.872	−11.852	−19.800	−87.45	−66.23	−21.22

VDW, van der Waals

^a^ Compound source: ChemSpider (https://www.chemspider.com/, accessed 10 Feb 2024).

### Bobcat339 suppressed SARS-CoV-2 infection and inhibited N-protein expression in hiPSC-CMs

**Fig. 6.**
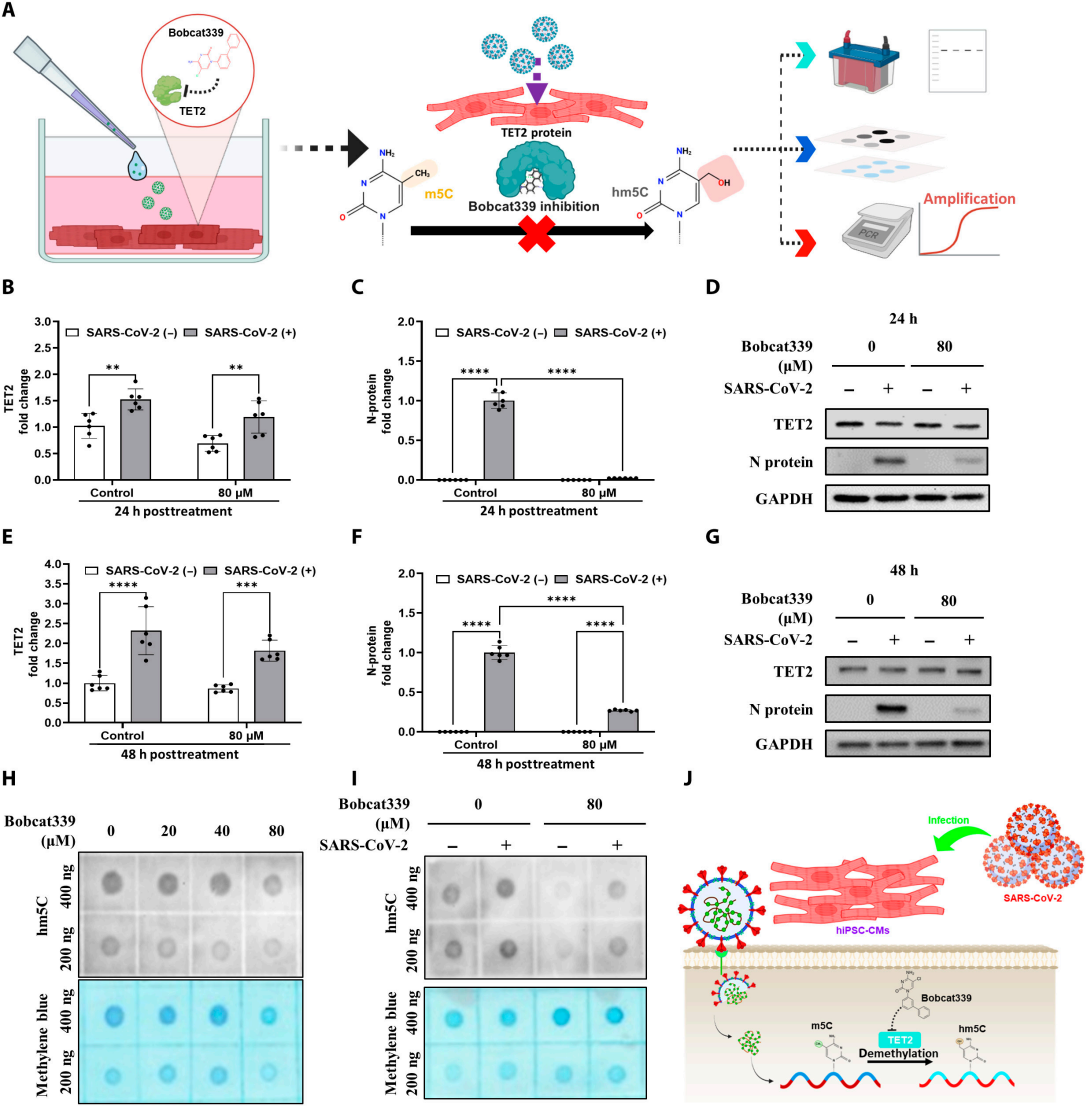
Treatment with Bobcat339 suppressed the viral replication and N-protein expression in infected hiPSC-CMs during SARS-CoV-2 infection. (A) Experimental workflow for assessing virus-mediated regulation of TET2 and its functional role using the selective TET2 inhibitor Bobcat339. (B) qRT-PCR analysis of TET2 mRNA expression in SARS-CoV-2-infected hiPSC-CMs treated with Bobcat339 (80 μM) or dimethyl sulfoxide (DMSO) for 24 h. (C) qRT-PCR analysis of N-protein mRNA expression in SARS-CoV-2-infected hiPSC-CMs treated with Bobcat339 (80 μM) or DMSO for 24 h. (D) Western blot analysis showing the protein expression levels of TET2 and SARS-CoV-2 N protein in hiPSC-CMs treated with Bobcat339 or DMSO for 24 h postinfection. (E) qRT-PCR analysis of TET2 mRNA expression in hiPSC-CMs treated with Bobcat339 or DMSO for 48 h postinfection. (F) qRT-PCR analysis of N-protein mRNA expression in hiPSC-CMs treated with Bobcat339 or DMSO for 48 h postinfection. (G) Western blot analysis showing the protein expression levels of TET2 and SARS-CoV-2 N protein in hiPSC-CMs treated with Bobcat339 or DMSO for 48 h postinfection. (H) Dot blot analysis of total RNA extracted from hiPSC-CMs showing hm5C levels after treatment with different concentrations of Bobcat339 (0 to 80 μM) or DMSO. Methylene blue (MB) was used as a loading control. (I) Dot blot analysis of total RNA extracted from hiPSC-CMs showing hm5C levels after treatment with Bobcat339 (80 μM) or DMSO, with or without SARS-CoV-2 infection. MB was used as a loading control. (J) Schematic diagram illustrating the proposed mechanism: SARS-CoV-2 infects hiPSC-CMs and induces TET2-mediated RNA demethylation (conversion of m5C to hm5C), which is inhibited by Bobcat339. Data in panels (B), (C), (E), and (F) are presented as mean ± SEM from 3 independent experiments. Statistical significance: **P < 0.01 and ****P < 0.0001.

The family of TET methylcytosine dioxygenases (TET1, TET2, and TET3) has been shown to catalyze the hydroxylation of m5C to generate hm5C [20]. Bobcat339, a cytosine-based TET2-specific inhibitor, was shown to block the enzymatic activities of both TET1 and TET2 [33]. Based on the TET2-knockdown results in Fig. 4, we proposed that inhibition of TET2 enzyme activity by the TET inhibitor Bobcat339 could further suppress the replication of SARS-CoV-2 in infected hiPSC-CMs (Fig. 6A). We therefore investigated the antiviral potential of Bobcat339 involved in the biomolecular mechanism of TET2 inhibition and epitranscriptomic modulation of SARS-CoV-2 infection. At the transcript level, TET2 mRNA expression was not significantly affected by Bobcat339 treatment at 24 h post-SARS-CoV-2 infection (Fig. 6B). Furthermore, the qRT-PCR results revealed that the mRNA levels of N protein were significantly decreased in the Bobcat339-treated hiPSC-CMs at 24 h postinfection (Fig. 6C). Notably, our western-blotting findings revealed that Bobcat339 treatment significantly decreased the protein expression levels of N protein but did not affect TET2 protein expression in infected hiPSC-CMs at 24 h postinfection (Fig. 6D). In addition, consistent with the findings at 24 h postinfection, at the transcript level, TET2 mRNA expression was not significantly affected by Bobcat339 treatment at 48 h postinfection (Fig. 6E), whereas N-protein mRNA levels were clearly reduced under the same conditions (Fig. 6F). At 48 h postinfection, the immunostaining results revealed that N-protein expression persisted in the infected group, whereas the signal showed a large reduction in the Bobcat339-treated group (Fig. 6G). However, Bobcat339 treatment did not affect TET2 protein expression in infected hiPSC-CMs (Fig. 6G). These findings suggest that treatment with Bobcat339 effectively suppresses SARS-CoV-2 replication and blocks N-protein expression partly through the inhibition of TET2 activity.

Bobcat339 is a newly developed and synthesized cytosine-based TET2 enzyme inhibitor that competes with the m5C substrate [34,35]. Bobcat339 inhibits TET2 demethylase activities by competitively inhibiting cytosine-derived molecules that fit into the dioxygenase active site of TET2 [18]. Our findings revealed that Bobcat339 suppressed SARS-CoV-2 infection and inhibited N-protein expression in hiPSC-CMs. We further propose that Bobcat339-associated anti-SARS-CoV-2 infection may involve TET2 inhibition to modulate RNA modification. First, our drug evaluation revealed that treatment with increasing concentrations of Bobcat339 (20 to 80 μM) dose-dependently reduced hm5C RNA levels, with 80 μM showing a clearly observable inhibitory effect (Fig. 6H). Moreover, the results of the cell viability test revealed that cell morphology and growth were normal in hiPSC-CMs following treatment with 80 μM Bobcat339 (Fig. S2), supporting the use of this 80 μM concentration for subsequent experiments. To further evaluate the effects of Bobcat339 on RNA hydroxymethylation, RNA dot blotting was performed, and the results revealed that 80 μM Bobcat339 significantly suppressed hm5C levels in SARS-CoV-2-infected hiPSC-CMs (Fig. 6I); this is evidenced by a decrease in hm5C levels and a concurrent suppression of N-protein expression at both the RNA and protein levels (Fig. 6J). Moreover, the biomolecular roles of TET inhibitors and Bobcat339 in the epitranscriptomic regulation of RNA modifications during SARS-CoV-2 infection and N-protein replication should be further studied in the future (Fig. 6J).

### Computational protein–ligand binding prediction of Bobcat339 with molecular docking modeling of SARS-CoV-2 proteins

**Fig. 7.**
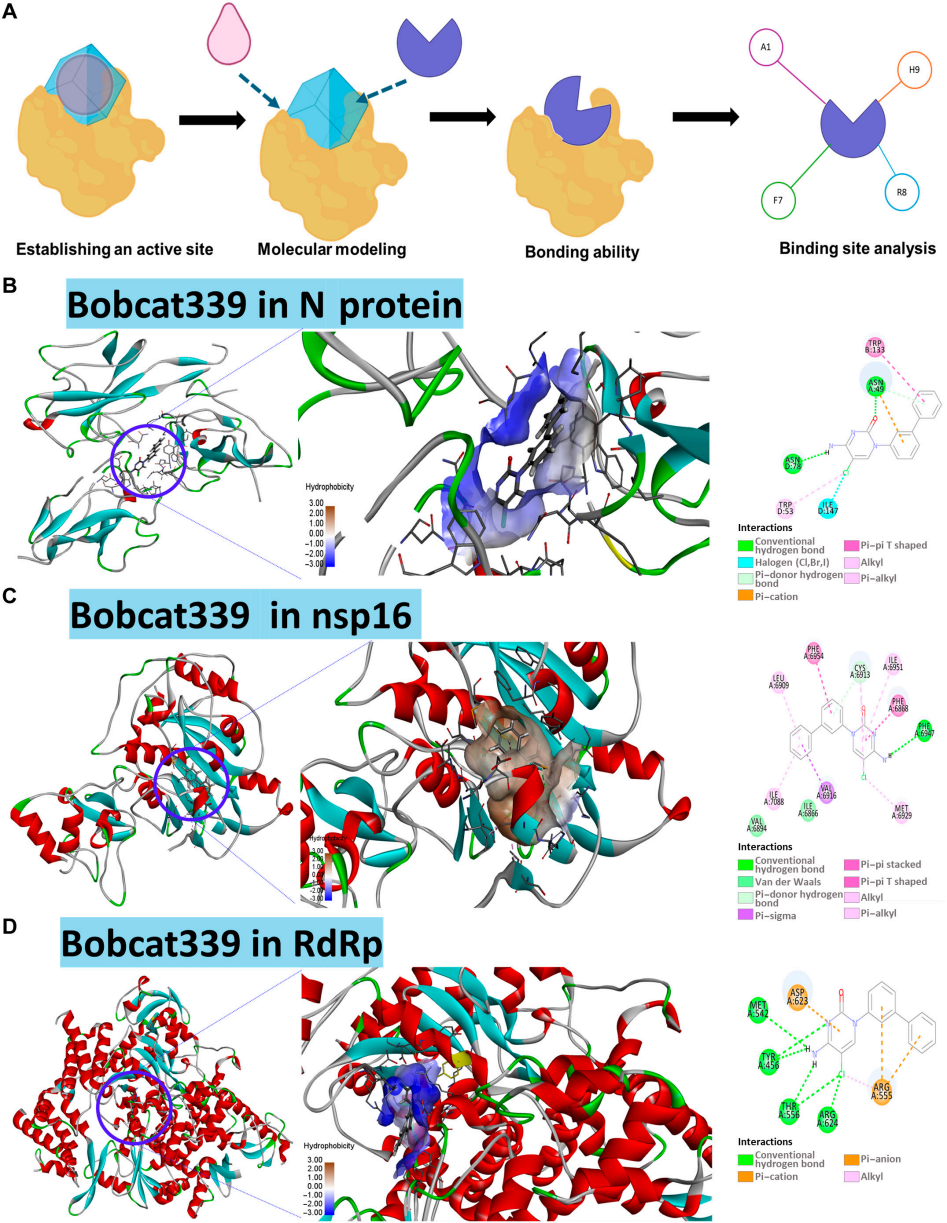
Computational protein–ligand binding prediction of Bobcat339 with molecular docking modeling of SARS-CoV-2 proteins in N protein, RNA-dependent RNA polymerase (RdRp), and nonstructural protein 16 (nsp16). (A) Illustration of the docking process. The diagram shows the process of target molecule docking by first defining the active center within the protein, then docking the ligand (Bobcat339) into the binding pocket, and finally observing the key interactions formed after docking, such as hydrogen bonds and hydrophobic contacts. (B) The docking of Bobcat339 into the RNA binding site of the SARS-CoV-2 N protein. The left panel depicts Bobcat339 positioned inside the binding pocket, while the middle panel highlights the hydrophobicity and detailed docking interactions. The right panel presents the specific molecular interactions between Bobcat339 and N-protein amino acid residues, including π–π stacking and hydrogen bonds. (C) The docking of Bobcat339 within the RNA binding domain of the SARS-CoV-2 nsp16 protein. The left panel presents the docking site; the middle panel, the binding pocket environment; and the right panel, interactions such as hydrogen bonds and hydrophobic contacts between Bobcat339 and surrounding amino acids. (D) Bobcat339 is docked into the RNA binding site of the SARS-CoV-2 RdRp. The left panel shows the overall docking location, the middle panel zooms into the binding pocket illustrating the hydrophobic interactions, and the right panel details the hydrogen bonding and hydrophobic interactions between Bobcat339 and specific RdRp residues.

As shown in Fig. 6, Bobcat339, a TET-specific enzymatic inhibitor, has been shown to preserve the potential against SARS-CoV-2 infection. We thought that the ability of Bobcat339 to prevent the infection and replication of SARS-CoV-2 not only followed the TET2-hm5C pathway but also directly blocked the functions of several crucial SARS-CoV-2 proteins. Therefore, we further explored the possible protein–ligand prediction ability of Bobcat339 via molecular docking modeling of SARS-CoV-2 proteins (Fig. 7A). Moreover, Bobcat339 binds with N protein, a critical protein for RNA binding and viral assembly; it forms hydrogen bonds with ASN49 and ASN78, a halogen bond with ILE147, and pi–alkyl interactions with TRP53 and TRP133. These interactions may disrupt the ability of N protein to assemble new virions (Fig. 7B). Furthermore, Bobcat339 binds within the active site of nsp16 protein methyltransferase, which plays an essential role in immune evasion, forming hydrogen bonds and pi–pi interactions with key residues such as CYS6913, VAL6916, PHE6868, and PHE6954. These interactions suggest potential disruption of the methyltransferase activity required for viral mRNA capping (Fig. 7C). In addition, the viral RdRp forms conventional hydrogen bonds with Bobcat339 through ASP537, ARG555, and HIS439 (Fig. 7D). In addition, pi–cation, pi–anion, and alkyl interactions with ARG555 and ASP623 stabilize its binding, suggesting possible interference with viral RNA replication.

**Fig. 8.**
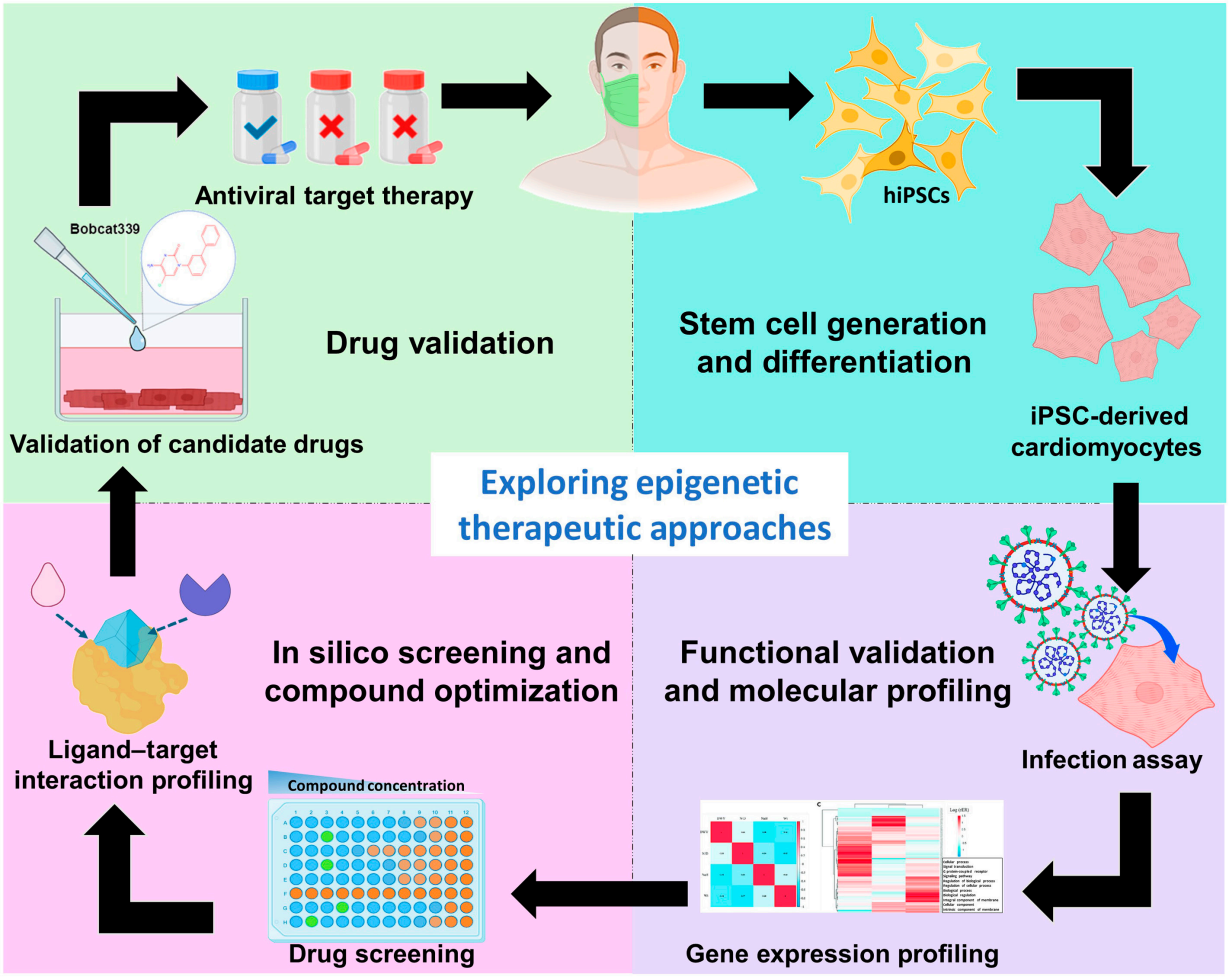
Development of epitranscriptomic-based drug screening and TET2-targeting pathways/TET inhibitors as a novel strategy for anti-SARS-CoV-2 infection via an in vitro model of hiPSC-derived cardiomyocytes. This schematic depicts the development of epigenetic therapies for SARS-CoV-2. hiPSCs are differentiated into cardiomyocytes, infected with SARS-CoV-2, and analyzed using NGS for molecular insights. Drug screening, computational evaluation, and validation with compounds like Bobcat339 aid in the advancement of antiviral therapy. This approach may significantly impact the future of antiviral drug development and personalized medicine, offering a sophisticated tool for understanding and combating systemic SARS-CoV-2 infections.

Based on these computational protein–ligand binding predictions of Bobcat339 with SARS-CoV-2 proteins (Fig. 7), we further utilized advanced molecular docking tools, DockThor [36] and GEMDOCK [37], to verify the docking energy between Bobcat339 and the target proteins. The analytical results validating the interactions of Bobcat339 with 3 SARS-CoV-2 proteins—N protein, nsp16, and RdRp—are presented in Table 2, based on docking analyses performed using DockThor and iGEMDOCK. Among the targets, Bobcat339 showed relatively better binding with nsp16 (PDB: 8F4Y) [38], as predicted by DockThor with a binding affinity of −9.716 kcal/mol and by iGEMDOCK with a total energy of −96.7333 kJ/mol, indicating a comparatively higher inhibitory potential. In summary, our study revealed that TET2 inhibition can efficiently suppress SARS-CoV-2 replication, viral protein assembly, and infectious activity in an in vitro infectious hiPSC-CM model. Notably, the simulation prediction analyses of our molecular docking models suggested that Bobcat339 functions as a multifaceted inhibitor to suppress both the function of TET2 (Figs. 5 and 6) and the replication of SARS-CoV-2 via the inhibition of the activities of the nsp16, RdRp, and N proteins (Fig. 7 and Table 2). Overall, our study revealed that TET2 and SARS-CoV-2 presented positive feedback to increase SARS-CoV-2 replication and infection efficiency in an in vitro epigenetic drug screening of a hiPSC-CM model (Fig. 8).

**Table 2.** Computational evaluation of Bobcat339 multitargeted binding docking with SARS-CoV-2 proteins via DockThor and iGEMDOCK. For docking simulations, (a) the DockThor software (https://dockthor.lncc.br/v2/, accessed 10 Aug 2024) was used to prepare ligand and receptor files, adhering to previously published molecular docking protocols, and (b) iGEMDOCK provided complementary docking analysis, offering detailed interaction energies including hydrogen bond contributions.

Protein (PDB ^a^)	(a) DockThor				(b) iGEMDOCK		
	Affinity (kcal mol^−1^)	Total energy (kcal mol^−1^)	VDW energy (kcal mol^−1^)	Elec. energy (kcal mol^−1^)	Total energy (kJ mol^−1^)	VDW energy (kJ mol^−1^)	H bond (kJ mol^−1^)
TET2 (7NE3)	−7.968	38.928	−8.901	−7.360	−101.03	−82.41	−18.62
N protein (8IV3)	−7.755	23.316	−23.429	−7.942	−66.8758	−62.076	−4.79976
nsp16 (8F4Y)	−9.716	20.863	−27.224	−6.770	−96.7333	−84.9124	−11.8209
RdRp (7BV2)	−8.331	27.427	−13.248	−14.228	−86.4503	−48.9121	−37.5382

^a^ PDB: Protein Data Bank (https://www.rcsb.org/, accessed 9 Aug 2024).

## Discussion

The COVID-19 pandemic has created significant health and biomedical challenges, with SARS-CoV-2 affecting the circulation system and leading to severe cardiac complications, including myocarditis and cardiomyocyte necrosis [39]. The technological platforms of hiPSC-differentiated lineages and hiPSC-CMs provide valuable models for studying SARS-CoV-2 infections and developing novel infectious therapeutic strategies [5,6,8,40]. The platform of hiPSC-CMs has been used as an in vitro SARS-CoV-2 infection model to determine the inflammatory process involved in the ability of TNF-α to exacerbate the cytokine storm and aggravate myocardial damage in patients with COVID-19 [11]. Furthermore, iPSC-derived lung organoids have been used to model human respiratory physiology, enabling drug screening and demonstrating that lipid-modifying drugs can inhibit SARS-CoV-2 entry and replication [7]. In this study, to comprehensively understand the pathogenic molecular mechanisms of epigenetic and epitranscriptomic expression in SARS-CoV-2 infection, an in vitro SARS-CoV-2-infected hiPSC-CM model was used to assess the pivotal role of TET2-mediated epitranscriptomics and RNA modification in the replication and infection process of SARS-CoV-2 (Figs. 2 to 4). We demonstrated that silencing TET2 effectively disrupted the viral replication cycle, reducing both the production of viral RNA and N-protein expression (Fig. 4), highlighting the crucial role of TET2 in facilitating viral replication. Furthermore, TET2 knockdown significantly reduced the level of hm5C RNA methylation, as detected by dot-blotting analysis (Fig. 4). Under SARS-CoV-2 infection, the levels of hm5C were markedly suppressed by TET2-knockdown treatment in infected hiPSC-CMs (Fig. 4I). These results suggest that TET2 knockdown not only effectively suppresses viral infection with N-protein expression but also alters epitranscriptomics and RNA modification in SARS-CoV-2-associated cardiomyopathy. To explore TET2 as a potential therapeutic target, we performed molecular docking simulations to assess the binding profiles of several small-molecule inhibitors, including Bobcat339, TETi76, and TFMB-2HG. Docking these compounds with the TET2 catalytic domain in complex with DNA revealed distinct binding patterns with key catalytic residues, suggesting that Bobcat339 is a reference compound for targeting TET2 protein binding compared with other molecules (Fig. 5). Notably, our results revealed that the therapeutic potential of Bobcat339 (a TET enzymatic inhibitor) significantly suppressed viral replication and N-protein expression in infected hiPSC-CMs (Fig. 6). Importantly, computational molecular docking analyses validated strong binding affinities between Bobcat339 and key SARS-CoV-2 proteins, RdRp, nsp16, and N protein (Fig. 7). Complementary molecular docking analyses revealed strong protein interactions and binding affinities between Bobcat339 and key viral proteins, including RdRp, nsp16, and N protein (Fig. 7). Collectively, these findings highlight the importance of epitranscriptomics and RNA modification in the SARS-CoV-2 replication process and support the targeting of TET2-mediated epitranscriptomic pathways as a novel therapeutic strategy (Fig. 8).

The viral replication and infectious activity of SARS-CoV-2 infection rely on the viral RdRp for replication, making RdRp a well-established antiviral drug target [41]. Previous studies have demonstrated that nucleotide analogs, including cytosine analogs such as sofosbuvir, inhibit SARS-CoV-2 RdRp by terminating RNA synthesis [42]. These findings extend to other antiviral agents that target the viral polymerase, providing a molecular basis for their inhibitory effects. Specifically, the active triphosphate form of sofosbuvir is incorporated by SARS-CoV-2 RdRp, halting further RNA synthesis, a mechanism shared by several other nucleotide analogs [43]. Moreover, nsp16, a methyltransferase, plays a key role in the SARS-CoV-2 regulation of viral RNA stability, the viral life cycle, and immune evasion during host infection [44]. Inhibition of nsp16 activity can effectively disrupt viral propagation and strengthen the host immune response, serving as a potential therapeutic target against SARS-CoV-2 infection [45]. In addition, N protein is highly conserved and plays a vital role in viral assembly, the replication cycle, and infectivity [46]. Mutations in N protein have been reported in Delta, Omicron, and other variants of SARS-CoV-2, which are known to cause viral-mutated infectivity and drug resistance [47]. Therefore, how to screen candidates efficiently and develop multiple targeted therapeutics is still extremely critical for facing the challenges of SARS-CoV-2 variant infection and emergent infectious diseases. Based on our findings of TET2-mediated targeting (Figs. 3 and 4) and TET2 inhibitors’ drug efficacy (Fig. 6) in vitro in SARS-CoV-2-infected hiPSC-CMs and computational molecular docking analysis (Figs. 5 and 7), the identification of TET2-mediated hm5C methylation presents a broad spectrum of main infectious activities and may serve as novel anti-COVID-19 agents against host-infectious therapeutics for RNA-based emergent infectious diseases (Fig. 8).

Bobcat339 has been shown to have the pharmacological ability to suppress the abundance of hm5C in DNA by inhibiting TET activity, presenting a cytosine-based TET-selective inhibitor [35]. Previous findings have demonstrated that Bobcat339 is capable of specifically inhibiting the enzymatic activities of TET1 and TET2 in different disease models. A previous study confirmed that Bobcat339 can effectively degrade the TET3 protein and lead to increased feeding in a TET3-dependent manner, making it a potential clinical drug candidate for treating anorexia nervosa and stress-related disorders [48]. Recent reports further demonstrated that Bobcat339 can trigger TET3 degradation and deplete TET3-overexpressing human macrophage-induced chronic inflammatory processes, underscoring the therapeutic potential of targeting TET3 in macrophages as a strategy for managing endometriosis [49]. TET2 is known to modulate RNA methylation, influencing viral gene expression and replication. For example, TET2 has been shown to act against influenza A virus by hypomethylating interferon-associated promoters, thereby activating the innate immune response [50]. In this study, our findings revealed that TET2 inhibition plays a regulatory role in modulating the host response to suppress SARS-CoV-2 infection and N-protein expression (Fig. 4). Notably, treatment with Bobcat339, a TET2 inhibitor, effectively suppressed SARS-CoV-2 replication and N-protein expression in hiPSC-CMs (Fig. 6), even when administered postinfection. In addition, an in vitro confocal immunofluorescence survey revealed that the protein levels of hm5C, in parallel with increasing dsRNA expression, were markedly increased in infected hiPSC-CMs during SARS-CoV-2 infection (Fig. 3H). Furthermore, TET2 knockdown profoundly inhibited viral infectious activity, suppressed N-protein expression, and positively parallel decreased the level of hm5C during SARS-CoV-2 infection (Fig. 4). Following these crucial findings, our RNA dot-blotting analysis revealed that treatment with Bobcat339 markedly suppressed SARS-CoV-2 infection and significantly suppressed hm5C levels in SARS-CoV-2-infected hiPSC-CMs (Fig. 6). Overall, we demonstrated that TET2 inhibition can effectively suppress SARS-CoV-2 infection, and treatment with a TET2 inhibitor, Bobcat339, has the ability to decrease N-protein expression in SARS-CoV-2-infected hiPSC-CMs, suggesting attractive epitranscriptomics-driven therapeutic approaches for treating COVID-19 pandemic infection.

Using DockThor and iGEMDOCK as molecular docking analytic platforms, the findings of our computational protein–ligand binding identifications indicate that Bobcat339 can form stable interactions with multiple viral proteins (Fig. 7), supporting its ability to interfere with various stages of the SARS-CoV-2 life cycle (Table 1). Among these, a higher binding affinity was observed with the nsp16 protein, implicating Bobcat339 as a potential inhibitor of the methyltransferase activity essential for viral mRNA capping, a process critical for the virus to evade immune detection. Further analysis suggested that Bobcat339 may also interact with RdRp and TET2, proteins that play pivotal roles in viral RNA replication and host–virus interactions. These interactions could disrupt RNA synthesis and other vital viral processes. In support of this hypothesis, compound 5a, a structurally characterized covalent inhibitor of the nsp16–nsp10 complex, has been shown to bind selectively to cysteine residues within these proteins [38]. Notably, compound 5a covalently binds to the catalytic residues of nsp16 and nsp10 following extended incubation, suggesting that covalent modification may serve as an effective mechanism for potent inhibition. Utilizing the same PDB ID (PDB: 8F4Y) [38], our computational molecular docking results suggest that similar effects may be observed, potentially leading to covalent binding or inducing conformational changes that could enhance its antiviral efficacy (Fig. 8 and Table 2). While computational models offer valuable preliminary insights, it is imperative to conduct experimental validation to confirm these interactions and to clarify the specific mechanisms through which Bobcat339 may act as a multitarget inhibitor against SARS-CoV-2. Furthermore, targeting TET2-associated pathways and TET inhibitors may represent a novel approach in drug development for the treatment of severe emerging infectious diseases, including SARS-CoV-2 infection (Fig. 8).

In conclusion, this study utilized an in vitro hiPSC-derived cardiomyocyte platform, providing a powerful model for epigenetic drug screening and identifying therapeutic targets against SARS-CoV-2-induced myocarditis and heart tissue damage. This innovative platform has the potential to revolutionize drug discovery and personalized medicine by developing COVID-19 patient-specific disease models and enabling efficient screening efficiency and efficacy with biosafety. This approach may significantly impact the future of antiviral drug development and personalized medicine, offering a sophisticated tool for understanding and combating systemic SARS-CoV-2 infections (Fig. 8). Overall, to our knowledge, we demonstrated that TET2-related epitranscriptomic modification and Bobcat339-targeting treatment constitute a pioneering epigenetics-based approach and protein-binding structure simulation-based strategy for discovering novel lead compounds and effective therapeutics for treating COVID-19 and new emergent life-threatening infections.

## Data Availability

The authors declare that all data supporting the findings of this study are available within the paper and its Supplementary Materials files.
