# Supplementary material for: Epitranscriptomic Modulation of TET2 Inhibition Suppressed SARS-CoV-2 Infection and Blocked Viral Nucleocapsid Protein in Induced-Pluripotent-Stem-Cell-Derived Cardiomyocyte Screening Models
Source: Biomater Res. 2025 Jul 22;29:0229. doi: 10.34133/bmr.0229 (PMC12280876; doi:10.34133/bmr.0229)

# Supplementary Figures


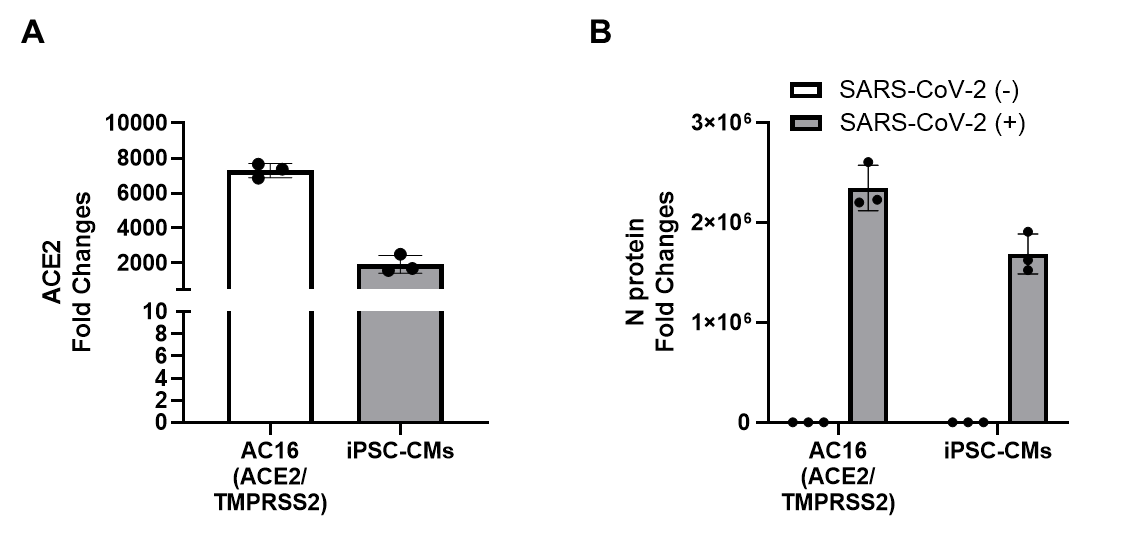


**Supplementary Figure 1. Engineering of ACE2/TMPRSS2-Expressing Human Cardiomyocytes for SARS-CoV-2 Infection Studies.** Stable expression of human ACE2 and TMPRSS2 in AC16 cells was established following a previously described approach. AC16 human cardiomyocyte cells were maintained in DMEM/F12 supplemented with 12.5% fetal bovine serum at 37°C in a humidified incubator with 5% CO₂. Cells stably expressing human ACE2 and TMPRSS2 were generated by lentiviral transduction and selected with 1 μg/ml puromycin and 1 μg/ml blasticidin. Following SARS-CoV-2 infection, ACE2 and viral nucleocapsid (N) mRNA levels were quantified by qPCR.


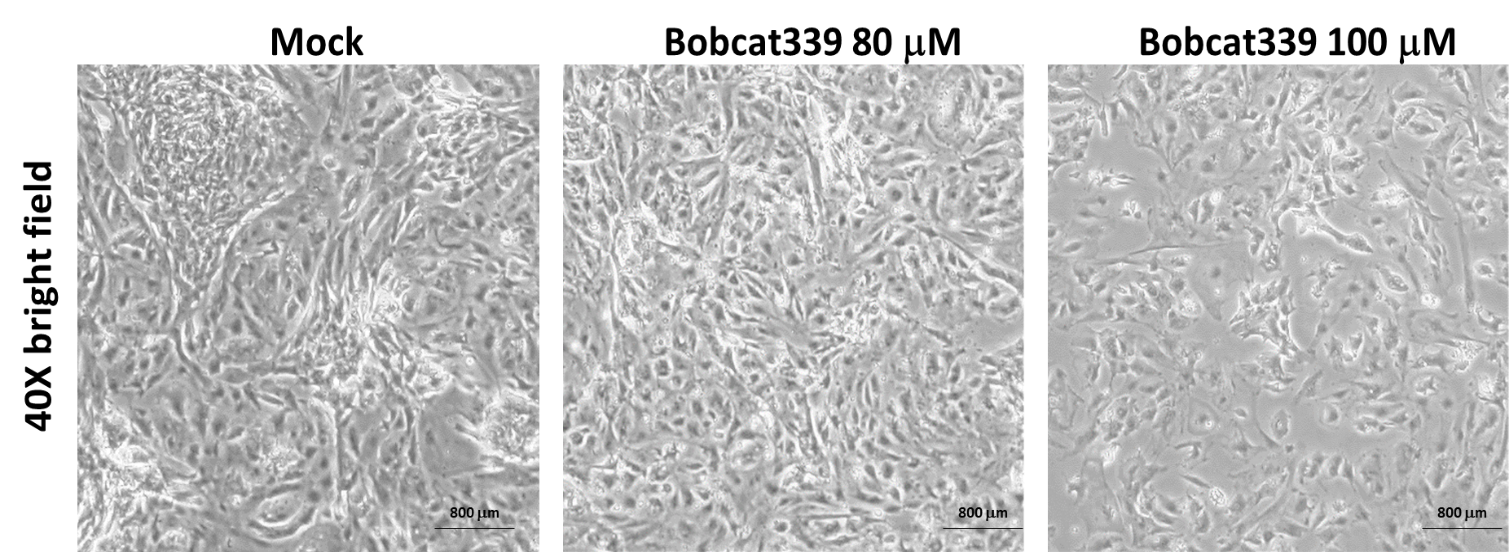


**Supplementary Figure 2**. **Morphological Effects of Bobcat339 on iPSC-Derived Cardiomyocytes.** Human induced pluripotent stem cells (hiPSCs) were differentiated into cardiomyocytes (hiPSC-CM) through standard differentiation protocols. After maturation, the cells were treated with the TET enzyme inhibitor Bobcat339 at concentrations ranging from 80 to 100 μM. Under brightfield microscopy, cells treated with 100 μM Bobcat339 showed marked morphological differences compared to the untreated control group. Specifically, the 100 μM concentration exhibited cytotoxic effects, including reduced cell density and abnormal morphology, indicating potential toxicity at this dosage.

**Supplementary tables**

**Table S1**. The primer sequences utilized for RT-qPCR analysis.


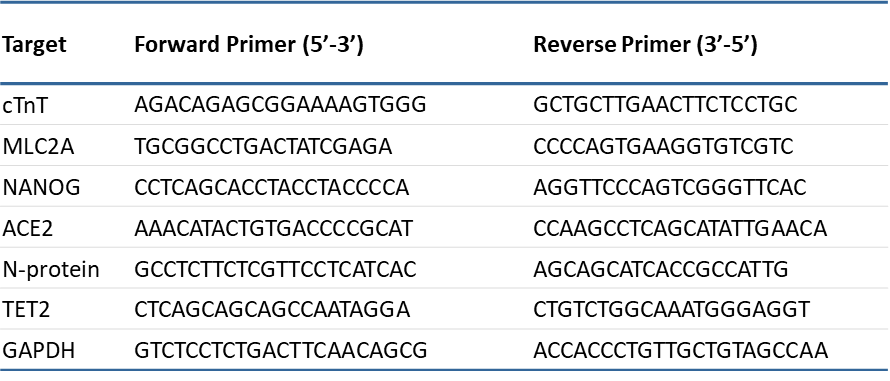


**Table S2**. List of antibodies.


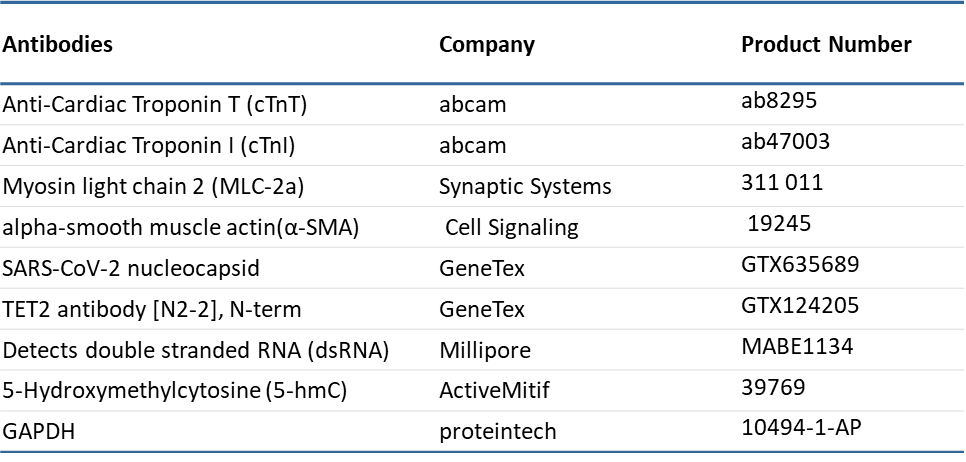

Supplement: Supplementary 1 — Figs. S1 and S2 Tables S1 and S2 [file bmr.0229.f1.docx]
